# Traditional uses, nutritional properties, phytochemical metabolites, pharmacological properties, and potential applications of *Lilium spp*.: a systematic review

**DOI:** 10.3389/fphar.2025.1713957

**Published:** 2025-11-24

**Authors:** Yi Wang, Huan Chen, Pucheng Feng, Deyun Wang, Xiaoquan Du

**Affiliations:** 1 School of Traditional Chinese Medicine, Shaanxi University of Chinese Medicine, Xianyang, China; 2 Department of Gastroenterology, The Affiliated Hospital of Shaanxi University of Chinese Medicine, Xianyang, China

**Keywords:** *Lilium spp.*, nutritional properties, chemical composition, anti-inflammatory properties, antidepressant effects, patent applications

## Abstract

**Introduction:**

*Lilium* spp., perennial bulbous plants native to the Northern Hemisphere, have long been valued in traditional medicine, particularly across Asia. The bulbs of *Lilium brownii* (“Bai He” in traditional Chinese medicine) have been documented since the Han dynasty as both food and medicine to nourish yin, moisten the lungs, clear heart fire, and calm the spirit—traditionally used for conditions such as depression and diabetes. Contemporary research has increasingly validated these traditional claims, revealing diverse pharmacological activities including antidepressant and antitumor effects.

**Methods:**

A comprehensive literature review was conducted using databases including Web of Science, PubMed, ACS Publications, Google Scholar, Baidu Scholar, and CNKI, as well as the *Encyclopedia of Life*, *Flora of China*, and *Plants of the World Online*. Taxa recorded in the *Chinese Pharmacopoeia* (2025) were included: *Lilium lancifolium* Thunb., *Lilium brownii* F. E. Brown var. *viridulum* Baker, and *Lilium pumilum* DC, and related species. All relevant multilingual publications were critically evaluated and accurately cited. Chemical structures of isolated metabolites were visualized using ChemDraw v19.0.

**Results:**

*Lilium* spp. are consumed in various culinary and processed forms, including steamed bulbs, flour, wine, and functional beverages. Nutritionally, they are rich in polysaccharides, saponins, dietary fibers, vitamins, amino acids, starch, pectin, phospholipids, and essential minerals such as calcium and iron. To date, 123 chemical metabolites have been isolated and characterized, with saponins, flavonoids, phenylpropanoids, and polysaccharides recognized as the principal bioactive metabolites. Pharmacological studies have demonstrated a wide range of biological activities-anti-inflammatory, antioxidant, antitumor, antidepressant, sedative, hepatoprotective, hypoglycemic, joint-protective, and immunomodulatory-observed in both *in vitro* and *in vivo* models.

**Discussion:**

*Lilium* spp. represent a valuable traditional medicinal and nutritional resource with promising potential for modern therapeutic and functional applications. Their integration into health products and cosmetics continues to expand; however, clinical validation remains limited. Further well-designed clinical trials are required to confirm the efficacy, safety, and mechanisms of *Lilium*-derived preparations. This review highlights recent advances to support the continued scientific and industrial development of *Lilium* as a multifaceted natural resource.

## Graphical Abstract

**Figure:**
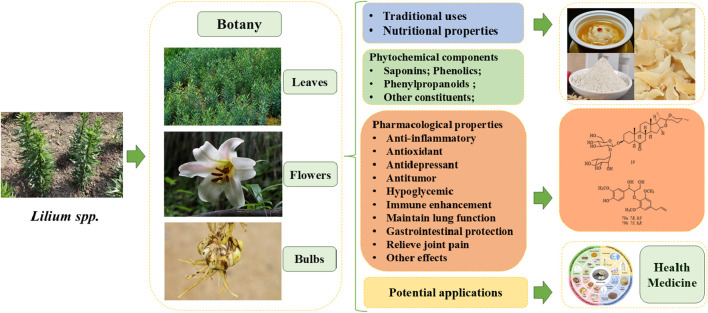

## Highlights

- As a genus of perennial herbaceouds bulbous plants, *Lilium* spp. is widely distributed across Asia, North America, and Europe.
- This review summarizes the traditional uses, botanical descriptions, nutritional properties, pharmacological studies, and patent associated with the genus Lilium spp.
- The primary metabolites and characteristic compounds of *Lilium spp.* were well highlighted.
- The main biological activities and related material basis as well as underlying mechanisms of Lilium spp. were outlined.
- Future perspectives and challenges of *Lilium* spp. were proposed.

## 1 Introduction

*Lilium* spp., members of the family Liliaceae, are perennial herbaceous bulbous plants primarily native to China and widely distributed across the temperate regions of the Northern Hemisphere. They are extensively cultivated in East Asia, Europe, and North America, and are regarded as one of the most economically, horticulturally, and medicinally important plant genera worldwide (Chen et al., 2024). China is fully considered an important distribution center for *Lilium* spp., with 55 species having been reported to date (Chen X. et al., 2025). According to the Chinese Pharmacopoeia (2025 Edition), the bulbs of *Lilium lancifolium* Thunb. (LL), *L. brownii* F. E. Brown var. *viridulum* Baker (LB) and *L. pumilum* DC. (LP) are official sources of *Lilii Bulbus*, a traditional Chinese medicine used to treat palpitations, insomnia, and hemoptysis (Wei et al., 2024). Highly valued for its diverse biological activities, *Lilium* spp. is also increasingly regarded as a functional food. In traditional Chinese medicine (TCM), it is characterized as a cold-natured botanical drug with a sweet taste, and is used to moisten the lungs, clear heat, and calm the mind. Its applications include the treatment of cough, blood-tinged sputum, anxiety, insomnia, and disturbed sleep. The medicinal use of *Lilium* spp. in China dates back to the *Shen Nong Ben Cao Jing* (circa 25-220 AD), which documented the therapeutic properties of *L. brownii* viridulum (Tang et al., 2021).

Phytochemical investigations have revealed a diverse array of bioactive metabolites in *Lilium* spp., comprising polysaccharides, saponins, sterols, alkaloids, glycerolglycolipids, phenylpropanoids, flavonoids, and phenolic acids (Li C. H. et al., 2024). *Lilium* spp. are extensively cultivated worldwide owing to their remarkable ornamental, edible, and medicinal properties. According to the Encyclopedia of Traditional Chinese Medicine (1985), lily bulbs contain various bioactive constituents, including colchicine, alkaloids, starch, proteins, and lipids. The anthers of *Lilium* longiflorum are particularly rich in carotenoids, with flavonoids accounting for approximately 91.7%–94.0% of the total pigments. Similarly, the anthers of *Lilium* lancifolium contain 2.68% moisture, 4.17% ash, 21.29% protein, 12.43% lipids, 3.61% starch, and 11.47% reducing sugars, along with vitamins B_1_, B_2_, B_6_, vitamin C, and β-carotene (Liu X. Y. et al., 2025). As a member of the Liliaceae family, *Lilium* spp. is one of the primary botanical families used in skincare cosmetic formulations. Antioxidant and ultraviolet (UV) absorption capacities are important evaluation indices of cosmetic quality. Polyphenols (including phenolic acids, flavonoids, lignins, etc.) exhibit good UV absorption capacity due to their aromatic rings (Hutskalova and Sparr, 2025; Tang et al., 2022). Bulbs of *Lilium* spp. possess not only nutritional value but also medicinal properties (Wang M. et al., 2024). This potent traditional remedy is frequently employed in folk medicine for the effective treatment of various ailments, including, but not limited to, coughs, asthma, insomnia, and anxiety (Bao et al., 2024). In Japan, *Lilium* species are incorporated into culinary traditions and gourmet food production. Their petals are often used to garnish sushi and served during tea ceremonies. In Korea, lilies are also commonly utilized in food and beverage processing (Lupşor et al., 2025). Lily-derived polysaccharides have been increasingly applied in the development of functional foods and nutritional supplements. Owing to their abundance of essential amino acids and trace elements, these polysaccharides can be incorporated into various food products to enhance nutritional value and improve overall dietary profiles. For instance, lily polysaccharides have been utilized in the formulation of composite protein beverages (Chen X. et al., 2025). In the pharmaceutical field, lily polysaccharides can serve as immunomodulators, offering adjuvant therapy to patients with immunodeficiency or immune abnormalities (Wang M. et al., 2024).

In recent years, extensive phytochemical investigations have demonstrated that *Lilium* spp. are characterized by high levels of saponins, phenols, and phenylpropanoids. Saponins, which are widely distributed throughout the plant kingdom, have attracted increasing attention in the food, cosmetic, and pharmaceutical industries due to their distinctive physicochemical properties and diverse biological activities, including anticancer and cholesterol-lowering effects (Wang M. et al., 2024). Moreover, pharmacological studies have revealed that crude extracts and purified metabolites from different organs of *Lilium* spp. possess a broad spectrum of bioactivities, such as antidiabetic, sedative, anticancer, anti-inflammatory, antioxidant, hepatoprotective, anti-obesity, gut-protective, and antidepressant effects. These pharmacological findings are consistent with the traditional medicinal and nutritional applications of the genus. Consequently, *Lilium* species have recently expanded their utilization beyond traditional medicine and food into the cosmetic and functional product industries (An et al., 2025).

In previous reviews, Zhou et al. systematically summarized the traditional uses, phytochemistry, and pharmacological properties of *Lilium* spp. species, providing a foundation for their potential applications in medicinal, food, and industrial fields (Zhou et al., 2021). More recently, Wang et al. focused on the nutraceutical aspects of *Lilium brownii* (Baihe), highlighting its bioactive metabolites and therapeutic effects (Wang Y. F. et al., 2024). Despite these efforts, several limitations persist, including insufficient coverage of nutritional profiles, emerging pharmacological mechanisms, and practical applications such as patents. Specifically, Zhou et al. lack recent discoveries post-2021, such as newly isolated metabolites and activities like anti-insomnia and hepatoprotection. Although timely, Wang et al. offer a brief account of phytochemistry and pharmacology with limited mechanistic insight and no discussion of patents or commercial uses. To address these research gaps, this review provides a comprehensive and up-to-date synthesis of the botanical characteristics, ethnopharmacological uses, nutritional composition, phytochemical diversity, pharmacological progress, patent landscape, and practical applications of *Lilium* species documented in the Chinese Pharmacopoeia. Particular emphasis is placed on newly characterized bioactive metabolites and recent mechanistic insights into their anti-insomnia and hepatoprotective activities. Furthermore, a systematic analysis of relevant patents is conducted to bridge the gap between academic research and industrial application. Collectively, this review refines the existing research framework and proposes prospective directions for future development.

## 2 Botanical description, geographic distribution, and taxonomy

### 2.1 Botanical description

**FIGURE 1:**
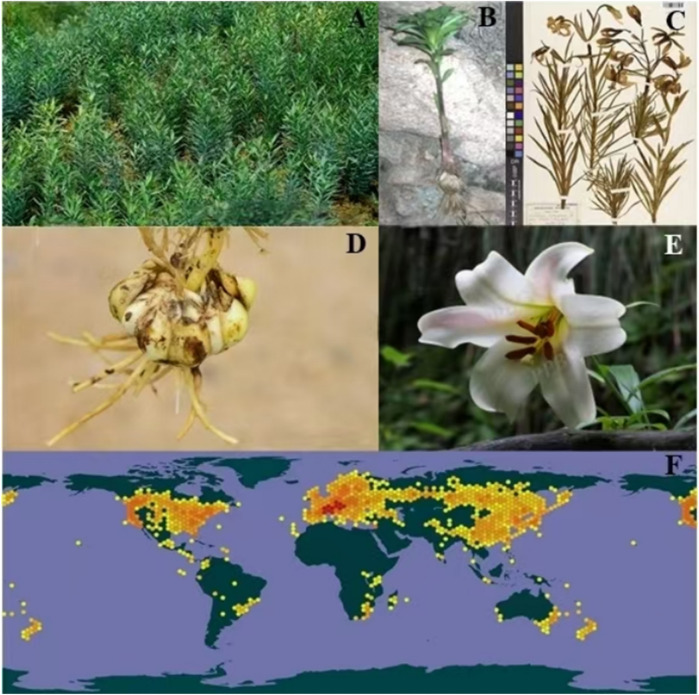
The growing habit **(A)** the morphology of plants **(B)** the plant specimen **(C)** the bulb of plants **(D)** the flowers **(E)** the distribution diagram worldwide **(F)** of the *Lilium* spp. (https://www.gbif.org/occurrence/4062681685; https://ppbc.iplant.cn/sp/41183; https://cn.bing.com/images/search?q=%E7%99%BE%E5%90%88&form=HDRSC2&first=1).

Botanically, *Lilium* spp., as showed in Figure 1B, is a genus of perennial herbaceous bulbous plants, characterized by ovoid or nearly spherical bulbs (Figure 1D). The bulbs possess numerous fleshy scales, which are ovate or lanceolate in shape, jointless or jointed, and typically white, though rarely yellow (Figure 1C). The stem is cylindrical, exhibiting small papillae either present or absent, with some varieties displaying purple stripes. Leaves are usually scattered, less often whorled, and come in various forms such as lanceolate, oblong-lanceolate, oblong-oblanceolate, elliptic, or linear. They are sessile or have short petioles, with entire margins or small papillae. The flower can be solitary or arranged in a raceme, and are rarely subumbellate or corymbose. Bracts are leaf-like but smaller in size (Figure 1A). Flowers are often brightly colored and sometimes fragrant (Figure 1E). There are six tepals arranged in two whorls, free and often somewhat connivent, forming a trumpet-shaped or campanulate structure, and rarely strongly recurved, usually lanceolate or spatulate. A nectary is present at the base, which may bear papillae on both sides, and some also possess a cristate or fimbriate appendage. The six stamens have subulate filaments that are pubescent or glabrous. Anthers are elliptic, dorsifixed, and T-shaped. The ovary is cylindrical, and the style is generally slender. The stigma is enlarged and three-lobed. Capsules are oblong and loculicidal. Seeds are numerous, flattened, and winged around (Flora of China Editorial Committee, 2015).

### 2.2 Geographic distribution

Globally, it is estimated that there are approximately 100–175 species of wild *Lilium* spp., primarily distributed throughout the Northern Hemisphere, including Asia (approximately 70 species), North America (approximately 24 species), and Europe (approximately 10–22 species). The eastern coast of Asia, the western coast of North America, and the Mediterranean region are particularly abundant in *Lilium* spp. resources (Figure 1F). As the center of natural distribution for *Lilium* spp. species, China boasts 36 original species and 15 varieties that are endemic to the country. Within China, *Lilium* spp. species are predominantly found in western Sichuan Province, northwestern Yunnan Province, southeastern Tibet Autonomous Region, and the northeastern regions (https://cn.vibaike.com/1260/).

### 2.3 Taxonomy

According to the 2025 edition of the Chinese Pharmacopoeia, the crude drug “Bulbus Lilii” is exclusively sourced from *Lilium* spp. taxonomically anchored in the kingdom *Plantae*, phylum *Streptophyta*, class *Equisetopsida*, subclass *Magnoliidae*, order *Liliales*, and family Liliaceae. The monograph unequivocally recognizes three botanical origins: (i) *Lilium lancifolium* Thunb., synonym *Lilium tigrinum*; (ii) *Lilium brownii* F. E. Brown var. *viridulum* Baker, synonyms *L. brownii* var. *viridulum* Baker, *Lilium brownii* var. *Ferum*, and *Lilium aduncum*; and (iii) *Lilium pumilum* DC., synonyms *Lilium pumilum* Redouté and *Lilium tenuifolium (* http://www.plantsoftheworldonline.org).

## 3 Traditional uses and nutritional properties

### 3.1 Traditional uses

Lily Bulbus was originally recorded in the *Shen Nong Ben Cao Jing*, and was listed as a medium grade Chinese medicine. It has the effects of nourishing yin, moistening the lung, clearing the heart, and calming the nerves. As a Chinese medicine for both medicine and food, Lily Bulbus has a long history of processing, dating back to the Han dynasty. *Lilium* spp. plants also have long been integral to traditional medicine systems across the globe, exhibiting extensive medicinal and culinary applications that reflect rich cross-cultural ethnopharmacological diversity. In China, *Lilium* spp. has been in use since the *Shen Nong Ben Cao Jing* of the Eastern Han Dynasty (25-220 AD), which documented the bulb of *L. brownii* viridulum as capable of “alleviating abdominal pain, promoting bowel movement, nourishing viscera, and clearing the heart to calm the mind” (Wang C. H. et al., 2018). Historical records reveal that in ancient times, different *Lilium* spp. species were often used interchangeably. Notably, *L. brownii var*. viridulum Baker, *L. lancifolium* Thunb., *L. pumilum DC*., and *L. concolor Salisb*. were all utilized for medicinal purposes (Wang C. H. et al., 2018). From the *Shen Nong Ben Cao Jing* to the *Ben Cao Meng Quan* of the Ming Dynasty (1,368–1,644), *L. brownii* F.E.Br. ex Miellez and its variant (var. viridulum) became established as the orthodox botanical origin. These were primarily sourced from authentic producing areas such as Jingzhou (Hubei), Cheng County (Gansu), Chuzhou (Anhui), and Heze (Shandong). Subsequent classic texts enriched the understanding of its efficacy. The *Ri Hua Zi Ben Cao* (circa 1,100) first recorded the medicinal use of *L. pumilum*. The *Ming Yi Bie Lu* (Han Dynasty, 220–450) and *Kai Bao Ben Cao* (circa 1,000) highlighted the non-toxicity of its bulb and its ability to reduce swelling and relieve pain. The *Ben Cao Jing Shu* (Ming Dynasty, 1,368–1,644) added the treatment of abdominal pain, heat-clearing, and bowel-movement-promoting functions to its portfolio. Li Shizhen’s *Ben Cao Gang Mu* (1,368–1,644) systematically distinguished *L. brownii* var. viridulum Baker, *L. lancifolium*, and *L. pumilum*. It established the legal status of all three as botanical origins, clarified that *L. brownii* var. viridulum was the authentic medicinal product, and refined the system of authentic producing areas (Zhang et al., 2019).

Building on this foundation, the modern *Chinese Pharmacopoeia* standardizes the use of bulbs from the aforementioned species, with a recommended daily dose of 6–12 g, for the treatment of palpitation, insomnia, and hemoptysis. It also includes the classical formula Baihe Dihuang Decoction (BDD), originally recorded in Zhang Zhongjing’s Synopsis of the Golden Chamber from the Eastern Han Dynasty. This decoction combines bulbs of *Lilium brownii* var. *viridulum* with roots of *Rehmannia glutinosa* Libosch. ex Fisch. et Mey. Contemporary studies have verified the efficacy of BDD in managing perimenopausal syndrome, insomnia, and pulmonary disorders (Jiang et al., 2019). Baihe Zhimu Decoction, a combination of bulbs from the same species with roots of Anemarrhena asphodeloides Bunge, is used clinically to address depression (Chen and Ding, 2012). The 2015 edition of the Pharmacopoeia further includes preparations such as Baihe Gujin Oral Liquid, thereby facilitating the transformation of traditional knowledge into standardized medications. The Chinese “medicine-food homology” system expands the scope of application. The Compendium of Materia Medica records that fresh bulbs can be steamed, cooked, consumed directly, or processed into powder. In summer, lily soup or porridge (sweetened with sugar) is consumed to relieve summer heat and moisten the lungs, leading to the creation of medicinal dishes such as stir-fried lily with celery. The core therapeutic principle of “nourishing Yin and moistening the lungs, clearing the heart and calming the mind” targets symptoms such as Yin-deficiency cough, hemoptysis, and insomnia associated with asthenia and vexation. This principle underlies the development of syndrome differentiation formulas such as Baihe Gujin Tang (BGD), indicated for lung and kidney Yin-deficiency syndromes, and Baihe Zhinue Tang (BZD), formulated for deficiency-heat with body fluid impairment syndromes (Chen and Ding, 2012). Furthermore, modern honey-processed *Lilium* spp. and Baihe Gujin Wan (2015 edition of the Pharmacopoeia) continue to enhance the development of clinical solutions for lung diseases.

Traditional medicine practices worldwide showcase regional specificity. For instance, North American indigenous tribes (Yurok/Karuk/Yana) utilize *L. pardalinum* Kellogg bulbs as a food source. They directly consume *Lilium* plants, prepared by steaming, baking, and other methods, as a source of carbohydrates and dietary fiber. (Munafo and Gianfagna, 2015). In Europe, *L. candidum* L. is used to treat trauma and mastitis. Folk practitioners often pound it and apply it to skin wounds and ulcers to promote wound healing; it is also used to address mastitis, particularly in lactating women. *L. martagon* L. is employed for liver diseases and can also alleviate symptoms such as dyspepsia and abdominal distension caused by mild hepatic damage. (Bruni et al., 1997; Pieroni, 2000). Japan uses L. *candidum* in burn treatment, and traditionally, it has also been employed there as a sedative, anti-inflammatory, antitussive, and general tonic botanical drug (Mimaki et al., 1999). And Korea employs *L. lancifolium* roots to address respiratory diseases (Lee et al., 2013). In India, *L. polyphyllum* D.Don bulbs are regarded as having galactagogue, expectorant, and tonic properties (Wang Y. F. et al., 2024). In summary, the “dual-use as medicine and food” tradition of *Lilium* spp. plants not only underscores the unique value of ethno-pharmacology but also offers a vital traditional knowledge base for modern active metabolite research and clinical translation.

Modern research on the genus *Lilium* centers on its core traditional uses, such as “nourishing Yin and moistening the lungs, clearing the heart, and calming the spirit.” Through pharmacological experiments and clinical studies, researchers have gradually elucidated the active metabolites and their mechanisms of action. Alkaloids, polysaccharides, steroidal saponins, and other metabolites (Sharifi-Rad et al., 2022) isolated from *Lilium* species have demonstrated clear anti-inflammatory, antioxidant, sedative-hypnotic, and lung-protective effects, aligning well with traditional Chinese medicine records for treating Yin-deficiency cough, insomnia, and palpitations. Significant progress has been made in modern research on the traditional applications of *Lilium* plants. Classic formulas such as Baihe Dihuang Tang and Baihe Zhimu Tang, recorded in the Chinese Pharmacopoeia, have been clinically validated to effectively alleviate perimenopausal syndrome, depression, and lung diseases (Fiorito et al., 2020).

### 3.2 Nutritional properties

In the natural world, there’s a plant with a bulb made up of dozens of white scales arranged like a lotus. Ancient people named it “Lily,” which means “a hundred pieces joined together.” Not only does the lily beautify nature with its elegant looks, but it also benefits human health thanks to its rich nutrients and medicinal properties. As early as 2002, the former Ministry of Health listed lily as one of the first “items that are both food and medicine,” showing the state’s long-term acknowledgment of its health benefits (Li and Liu, 2025). The lily, with its edible and medicinal dual attributes, holds a pivotal position in China’s food industry. Its dual-purpose utility was first recorded in the ancient Shen Nong’s Herbal Classic, making it a quintessential symbol of China’s traditional food and medicine heritage. Lily can be enjoyed in various culinary forms, from steaming and boiling as a fresh metabolite to being processed into products like lily powder, wine, and functional beverages. In traditional Chinese medicine, lily is esteemed for its abilities to alleviate phlegm and cough, nourish Yin and moisten the lungs, combat oxidative stress, modulate the immune system, and help regulate blood sugar.

Nutritionally, lily is a powerhouse, containing active bio-metabolites such as polysaccharides, saponins, and dietary fiber, along with essential nutrients like vitamins, amino acids, starch, pectin, and phospholipids, complemented by minerals including calcium and iron (Yu et al., 2024). Lily bulbs are a versatile metabolite that can be easily added to daily meals for their health benefits. For example, Lily Bulb and Lotus Seed Congee nourishes Yin and moistens the lungs, while Lily Bulb and White Fungus Soup enhances the complexion. According to traditional Chinese medicine, lily bulbs are also known for their skin-beautifying effects, thanks to their rich mucilage and various vitamins (Liu and Kang, 2025). In 1990, the Gansu Academy of Agricultural Sciences analyzed three key Chinese lily species (*Lilium* davidii, *Lilium* brownii var. viridulum, *Lilium* pumilum), finding protein (3.12%–3.36%), sucrose (3.67%–10.39%), reducing sugar (1.54%–3.00%), pectin (3.80%–5.61%), starch (11.10%–19.54%), fat (0.08%–0.18%), potassium (0.38%–0.64%), phosphorus (0.05%–0.07%), crude fiber (0.86%–1.11%), and ash (1.05%–1.35%). Lily bulbs are rich in nutrients, containing not only active metabolites such as polysaccharides (fresh weight 2.0–5.0g/100 g), steroidal saponins (fresh weight 0.1–0.3g/100 g), and dietary fiber (fresh weight 1.0–2.5g/100 g), but also abundant in vitamins, amino acids, starch (dry weight 11.10%–19.54%), and pectin (dry weight 3.80%–5.61%) (Ding et al., 2004). Modern medicine’s progress has spurred robust pharmacological research on lilies. Extracted active metabolites from lilies are driving the development of drugs and health products, especially for immune modulation and anti-tumor applications. Lily polysaccharide-based immunomodulators show promise in boosting immunity and treating diseases linked to low immune function, offering new treatment options for such patients. Meanwhile, research into lily-derived anti-tumor metabolites is ongoing, with the aim of creating innovative anti-cancer drugs that could significantly advance tumor treatment (Yuan, 2025). Lily is an economically efficient natural resource abundant in bioactive metabolites and nutrients, making it an ideal candidate for the development of functional foods with significant market potential. Meanwhile, as pharmacological research into lilies advances, active metabolites extracted from them are contributing to the development of drugs and health products (Zengin et al., 2022). Their efficacy has been particularly notable in immune regulation and anti-cancer fields, promising the development of new drugs that boost immunity and combat cancer (Sharifi Rad et al., 2014).

## 4 Phytochemical composition

**FIGURE 2:**
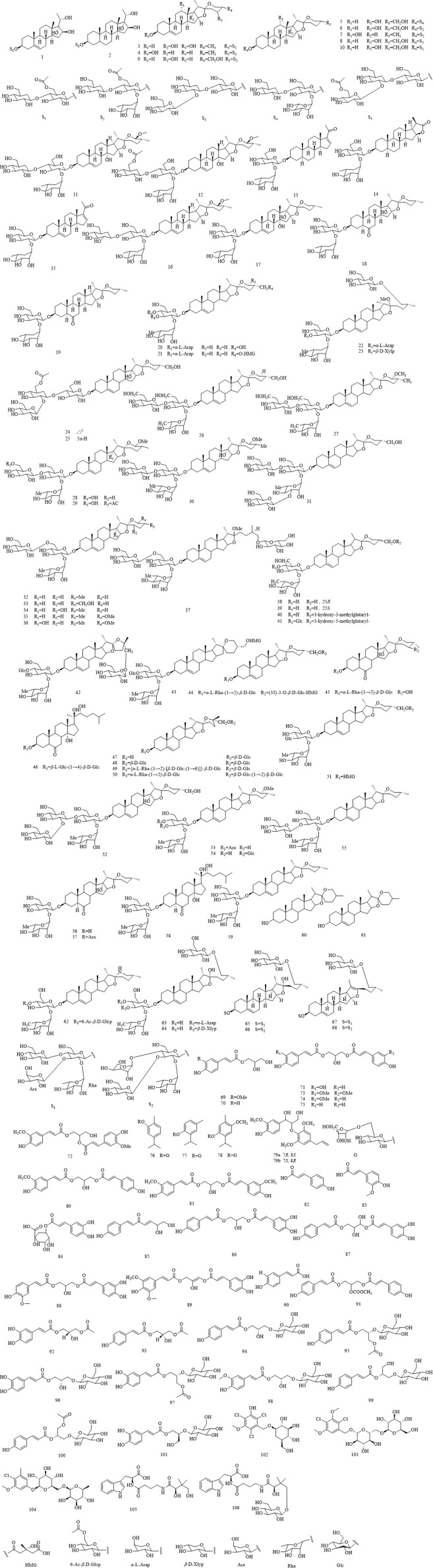
Chemical structures of metabolites isolated from the *Lilium spp*.

Currently, approximately **123** phytochemicals have been isolated and identified from various tissues of *Lilium* spp. (Table 1; Table 2). These mainly include saponins, phenols and phenylpropanoids, and other metabolites. Saponins, phenols, and phenylpropanoids are considered bioactive metabolites and are known to possess various pharmacological activities. The chemical metabolites that have been isolated and identified from the *Lilium* spp. are summarized in Table 1 and Table 2, while the structures of the major bioactive metabolites are illustrated in Figure 2.

**TABLE 1:** Chemical metabolites isolated and structurally identified from different medicinal parts of *Lilium spp*.

NO	Chemical metabolites	Molecular formula	Extracts	Parts	Source	Refs
Saponins						
1	(16*S*,17*R*,20*S*)-3*β*,16,17,20-tetrahydroxy-pregnane-5-ene-3-O-{*β*-Dglucopyranosyl-(1→4)-6-O-acetyl-*β*-D-glucopyranoside}	C_35_H_56_O_15_	EtOH	Bulbs	*L. lancifolium*	Liu et al. (2025b)
2	(16*S,*17*R,*20*S*)-3*β,*16*,*17*,*20-tetrahydroxy-5*α*-pregnane-3-O-{*β*-D-glucopyranosyl-(1→4)-6-Oacetyl-*β*-D-glucopyranoside}	C_35_H_58_O_15_	EtOH	Bulbs	*L. lancifolium*	Liu et al. (2025b)
3	(24*S,*25*S*)-3*β,*17*α,*24-trihydroxy-spirostan-5-ene-3-O-{O-*α*-L-rhamnopyranosyl-(1→2)-O-[6-O-acetyl-*β*-D-glucopyranosyl-(1→4)]-*β*-D-glucopyranoside}	C_47_H_74_O_20_	EtOH	Bulbs	*L. lancifolium*	Liu et al. (2025b)
4	(25*R*)-3*β*,12*α*-dihydroxy-spirostan-5-ene-3-O-{O-*α*-L-rhamnopyranosyl-(1→2)-O-[6-O-acetyl-*β*-D-glucopyranosyl-(1→4)]-*β*-D-glucopyranosid-e}	C_47_H_74_O_19_	EtOH	Bulbs	*L. lancifolium*	Liu et al. (2025b)
5	(25*S*)-3*β,*17*α,*27-trihydroxy-5*α*-spirostan-3-O-{O-*α*-L-rhamnopyranosyl-(1→2)-O-[*β*-D-glucopyranosyl-(1→4)]-*β*-D-glucopyranoside}	C_45_H_74_O_19_	EtOH	Bulbs	*L. lancifolium*	Liu et al. (2025b)
6	(25*S*)-3*β,*17*α,*27-trihydroxy-5*α*-spirostan-3-O-{O-*α*-L-rhamnopyranosyl-(1→2)-O-[6-O-acetyl-*β*-D-glucopyranosy-l-(1→4)]-*β*-D-glucopyranoside}	C_47_H_76_O_20_	EtOH	Bulbs	*L. lancifolium*	Liu et al. (2025b)
7	(25*R*)-3*β*,12*α*-dihydroxy-5*α*-spirostan-3-O-{O-*α*-L-rhamnopyranosyl-(1→2)-O [6-O-acetyl-*β*-D-glucopyranosyl-(1→4)]-*β*-D-glucopyranoside}	C_47_H_76_O_19_	EtOH	Bulbs	*L. lancifolium*	Liu et al. (2025b)
8	(25*S*)-3*β,*17*α,*27-trihydroxy-5*α*spirostan-3-O-{*β*-D-glucopyranoside-(1→4)-O-*β*-D-glucopyranosyl-(1→2)-O-6-O-acetyl-*β*-D-glucopyranoside}	C_47_H_76_O_21_	EtOH	Bulbs	*L. lancifolium*	Liu et al. (2025b)
9	(25*S*)-3*β,*17*α,*27-trihydroxy-spirostan-5-ene-3-O-{O-*α*-L-rhamnopyranosyl-(1→2)-O-[6-O-acetyl-*β*-D-glucopyranosyl-(1→4)]-*β*-D-glucopyranoside}	C_47_H_74_O_20_	EtOH	Bulbs	*L. lancifolium*	Liu et al. (2025b)
10	(25*S*)-5*α*-spirostane-3*β,*17*α,*27-triol 3-O-{O-*β*-D-glucopyranosyl (1→2)-O-*β*-D-glucopyranosyl-(1→4)-*β*-D-glucopyranoside	C_45_H_74_O_20_	EtOH	Bulbs	*L. lancifolium*	Liu et al. (2025b)
11	Lililancifoloside B	C_46_H_74_O_19_	EtOH	Bulbs	*L. lancifolium*	Zhou N. et al. (2024)
12	Lililancifoloside C	C_48_H_76_O_20_	EtOH	Bulbs	*L. lancifolium*	Zhou N. et al. (2024)
13	Lililancifoloside D	C_33_H_52_O_11_	EtOH	Bulbs	*L. lancifolium*	Zhou N. et al. (2024)
14	Lililancifoloside E	C_46_H_74_O_19_	EtOH	Bulbs	*L. lancifolium*	Zhou N. et al. (2024)
15	Spongipregnoloside A	C_34_H_52_O_10_	EtOH	Bulbs	*L. lancifolium*	Zhou N. et al. (2024)
16	(25*R,*26*R*)-26-methoxyspirost-5-en-3*β*-ol3-O-*α*-L-rhamnopyranosyl-(1→2)-O-[*β*-D-glucopyranosyl-(1→4)]-*β*-Dglucopyranoside	C_48_H_78_O_16_	EtOH	Bulbs	*L. lancifolium*	Zhou N. et al. (2024)
17	Ophiogenin3-O-*α*-L- rhanopyranosyl-(1→2)-*β*-D-glucopyranoside	C_40_H_64_O_13_	EtOH	Bulbs	*L. lancifolium*	Zhou N. et al. (2024)
18	(25*R*)-3*β,*17*α*-dihydroxy-5*α*- spirostan-6-one 3-O-*α*-L-rhamnopyranosyl-(l→2)-*β*-D-glucopyranoside	C_40_H_64_O_13_	EtOH	Bulbs	*L. lancifolium*	Zhou N. et al. (2024)
19	(25*R*) -3*β*-5*α*-spirostan-6-one 3-O-*α*-L-rhamnopyranosyl-(1→2)- *β*-D-glucopyranoside	C_40_H_64_O_12_	EtOH	Bulbs	*L. lancifolium*	Zhou N. et al. (2024)
20	(25*S*)-spirost-5-ene-3*β*,27-diol3-0-{0-x-L-rhamnopyranosyl-(1→2)-0-[x-L-arabinopyranosyl-(1→3)]-B-*β*-glucopy- ranoside}	C_44_H_70_O_17_	MeOH	Bulbs	*L. longiflorum*	Mimaki et al. (1994)
21	(25*R*)-27-0-[(*S*)-3-hydroxy-3-methylglutaryl]-spirost-5-ene-3*β*,27-diol3-0-{0-a-L-rhamnopyranosy-(1→2)-0-[a-L-arabinopyranosyl-(1–3)]-*β*-D-glucopyranoside}	C_50_H_73_O_19_	MeOH	Bulbs	*L. longiflorum*	Mimaki et al. (1994)
22	22-0-methy1-26-0-*β*-D-glucopyranosy-(25R)-furost-5-ene-3*β*,22ξ,26-triol3-0-{0-x-L-rhamnopyranosyl-(1→2)-0-[*α*-L-arabinopyranosy-(1→3)]-B-D-glucopyranoside}	C_52_H_77_O_22_	MeOH	Bulbs	*L. longiflorum*	Mimaki et al. (1994)
23	22-0-methy-26-0-*β*-D-glucopvranosyl-(25R)-furost-5-ene-3*β*,225,26-triol3-0-{0-a-L-rhamnopyranosy-(1-2)-0-[*β*-D-xylopyranosy-(1→3)-B-D-glucopyranoside}	C_52_H_77_O_22_	MeOH	Bulbs	*L. longiflorum*	Mimaki et al. (1994)
24	(25*S*)-spirost-5-ene-3*β*,17d,27-triol 3-O-{O-*β*-D-glu-(1→2)-O-*β*-D-glu-(1→4)-*β*-D-glu}	C_45_H_72_O_20_	MeOH	Bulbs	*L. martagon*	Satou et al. (1996)
25	(25*S*)-5*α*-spirostane-3*β*,17d,27-triol 3-O-{O-*β*-D-glu-(1→2)-O-*β*-D-glu-(1→4)-*β*-D-glu}	C_45_H_74_O_20_	MeOH	Bulbs	*L. martagon*	Satou et al. (1996)
26	(25*S*)-3*β*-{*β*-D-glucopyranosyl-(1→4)-[*α*-L-rhamnopyranosyl-(1→2)]-*β*-D-glucopyranosyloxy}spirost-5-en-27-ol	C_47_H_76_O_18_	EtOH	Bulbs	*L.* *candidum L*	Haladová et al. (1998)
27	(25*R*,26*R*)-3*β*-{*β*-D-glucopyranosyl-(1→4)-[*α*-L-rhamnopyranosyl-(1→2)]-*β*- d -glucopyranosyloxy}-26-methoxyspirost-5-ene	C_48_H_78_O_18_	EtOH	Bulbs	*L.* *candidum L*	Haladová et al. (1998)
28	(25*R*,26*R*)-26-methoxyspirost-5-ene-3*β*,17*α*-diol 3-0-{0-*α*-L-rhamnopyranos}l-(1→2)-0-[*β*-D-glucopyranosyl-(1→4)]*β*-D-glucopyranoside}	C_46_H_74_O_19_	EtOH	Bulbs	*L. candidum*	Mimaki et al. (1998)
29	(25*R*,26*R*)-26-methoxyspirost-5-ene-3*β*,17*α*-diol 3-0-{0-*α*-L-rhamnopyranosyl-(1→2)-0-[6-O-acetyl-*β*-D-glucopyranosyl-(1→4)]-*β*-D-glucopyranoside}	C_48_H_76_O_20_	EtOH	Bulbs	*L. candidum*	Mimaki et al. (1998)
30	(25*R*,26*R*)-26-methoxyspirost-5-ene-3*β*,17*α*-diol 3-0-{0-*α*-L-rhamnopyranosyl-(1→2)-*β*-D-glucopyranoside}	C_40_H_64_O_14_	EtOH	Bulbs	*L. candidum*	Mimaki et al. (1998)
31	(25*S*)-spirost-5-ene-3*β*,27-diol 3-0-{O-*β*-D-glucopy-ranosyl-(1→3)-O-*α*-L-rhamnopyranosyl-(1→2)-O-[*β*-D-glucopyranosyl-(1→4)]-*β*-D-glucopyranoside}	C_51_H_82_O_23_	EtOH	Bulbs	*L. candidum*	Mimaki et al. (1998)
32	(25*R*)-spirost-5-ene-3*β*-yl O-*α*-L-rhamnopyranosyl-(1→2)-O-[*β*-D-glucopyranosyl-(1→6)]-*β*-D-glucopyranoside	C_45_H_72_O_17_	MeOH	Bulbs	*L. candidum*	Mimaki et al. (1999)
33	(25*S*)-27-hydroxyspirost-5-ene-3*β*-yl O-*α*-L-rhamnopyranosyl-(1→2)-O-[*β*-Dglucopyranosyl-(1→6)]-*β*-D-glucopyranoside	C_45_H_72_O_18_	MeOH	Bulbs	*L. candidum*	Mimaki et al. (1999)
34	(23*S*,25*R*)-23-hydroxyspirost-5-ene-3*β*-yl O-*α*-L-rhamnopyranosyl-(1→2)-O-[*β*-D-glucopyranosyl-(1→6)]-*β*-D-glucopyranoside	C_45_H_72_O_18_	MeOH	Bulbs	*L. candidum*	Mimaki et al. (1999)
35	(25*R*,26*R*)-26-methoxyspirost-5-ene-3*β*-yl O-*α*-L-rhamnopyranosyl-(1→2)-O-[*β*-D-glucopyranosyl-(1→6)]-*β*-D-glucopyranoside	C_46_H_74_O_18_	MeOH	Bulbs	*L. candidum*	Mimaki et al. (1999)
36	(25*R*,26*R*)-17*α*-hydroxy-26-methoxyspirost-5-ene-3*β*-yl O-*α*-L-rhamnopyranosyl-(1→2)-O-[*β*-D-glucopyranosyl-(1→6)]-*β*-D-glucopyranoside	C_46_H_74_O_19_	MeOH	Bulbs	*L. candidum*	Mimaki et al. (1999)
37	(25*R*)-26-(*β*-D-glucopyranosyloxy)-22-methoxyfurost-5-en-3*β*-yl O-*α*-L-rhamnopyranosyl-(1→2)-O-[*β*-D-glucopyranosyl-(1→6)]-*β*-D-glucopyranoside	C_52_H_86_O_23_	MeOH	Bulbs	*L. candidum*	Mimaki et al. (1999)
38	(25*R*)-spirost-5-en-3*β*,27-ol 3-O-*α*-L- rhamnopyranosyl -(1→2)-*β*-D- glucopyranoside	C_39_H_63_O_13_	EtOH	Bulbs	*L. candidum*	Haladova et al. (2011)
39	(25*S*)-spirost-5-en-3*β*,27-ol 3-O-*α*-L- rhamnopyranosyl -(1→2)-*β*-D- glucopyranoside	C_39_H_63_O_13_	EtOH	Bulbs	*L. candidum*	Haladova et al. (2011)
40	(25*R*)-27-O-[(*S*)-3-hydroxy-3-methylylutaryl]-spirost-5-ene-3*β*,27-diol 3-O-*α*-L-rha-(1→2)-*β*-D- glucopyranoside	C_45_H_71_O_17_	EtOH	Bulbs	*L. candidum*	Haladova et al. (2011)
41	(25*R*)-27-O-[(*S*)-3-hydroxy-3-methylylutaryl]-spirost-5-ene-3*β*,27-diol3-O-{*α*-L-rha-(1→2)-O-[*β*-D-glu-(1→4)]-*β*-D- glucopyranoside }	C_51_H_81_O_22_	EtOH	Bulbs	*L. candidum*	Haladova et al. (2011)
42	26-O-*β*-D-glucopyranosylnuatigenin3-O-*α*-L-rhamnopyranosyl-(1→2)-O-[*β*-D-glucopyranosyl-(1→4)]-*β*-D-glucopyranoside	C_47_H_74_O_20_	MeOH	Bulbs	*L.brownii*	Mimaki and Sashida (1990)
43	27-O-(3-hydroxy-3-methylglutaroyl)spirost-5-ene-3*β*,27-diol (isonarthogenin)-3-O-*α*-L-rhamnopyranosyl-(1→2)-O-[*β*-D-glucopyranoside-(1→4)]-*β*-D-glucopyranoside	C_49_H_77018_	MeOH	Bulbs	*L.brownii*	Mimaki and Sashida (1990)
44	27-O-[(3*S*)-3-O-*β*-D-glucopyranosyl 3-methylglutaroyl]isonarthogenin 3-O-[*α*-L-rhamnopyranosyl-(1→2)]-*β*-D-glucopyranoside	C_51_H_80_O_22_	MeOH	Bulbs	*L. brownii*	Yang et al. (2012)
45	(24*S*,25*S*)-3*β*,17*α*,24-trihydroxy-5*α*-spirostan-6-one 3-O-[*α*-L-rhamnopyranosyl-(1→2)]-*β*-D-glucopyranoside	C_39_H_62_O_15_	MeOH	Bulbs	*L. brownii*	Yang et al. (2012)
46	Tenuifoliol 3-O-[*β*-D-glucopyranosyl-(1→4)]-*β*-D-glucopyranoside	C_39_H_66_O_15_	MeOH	Bulbs	*L. brownii*	Yang et al. (2012)
47	26-O-*β*-D-glucopyranosylnuatigenin	C_33_H_52_O_9_	MeOH	Bulbs	*L. brownii*	Yang et al. (2012)
48	26-O-*β*-D-glucopyranosylnuatigenin 3-O-*β*-D-glucopyranoside	C_39_H_62_O_14_	MeOH	Bulbs	*L. brownii*	Yang et al. (2012)
49	26-O-*β*-D-glucopyranosylnuatigenin 3-O-{*α*-L-rhamnopyranosyl-(1→2)-[*β*-D-glucopyranosyl-(1→6)]}-*β*-D-glucopyranoside	C_51_H_82_O_23_	MeOH	Bulbs	*L. brownii*	Yang et al. (2012)
50	26-O-[*β*-D-glucopyranosyl-(1→2)]-*β*-D-glucopyranosylnuatigenin 3-O-[*α*-L-rhamnopyranosyl-(1→2)]-*β*-D-glucopyranoside	C_51_H_82_O_23_	MeOH	Bulbs	*L. brownii*	Yang et al. (2012)
51	(25*R*)-27-O-[(S)-3-hydroxy-3-methylglutaroyl]-spirost-5-ene-3 *β*,27-diol 3-O-*α*-L-rhamnopyranosyl-(1→2)-O-[*β*-D-glucopyranosyl-(1→3)]- *β*-D-glucopyranoside	C_51_H_80_O_22_	MeOH	Bulbs	*L. regale*	Mimaki et al. (1993)
52	(25*S*)-spirost-5-ene-3 *β*,17 *α*,27-triol 3-O-*α*-L-rhamnopyranosyl-(1→2)-O-[*β*-D-glucopyranosyl-(1→2)-O- *β*-D-glucopyranosyl-(1→4)]-*β*-D-glucopyranoside	C_52_H_86_O_23_	MeOH	Bulbs	*L. regale*	Mimaki et al. (1993)
53	(25*R*,26*R*)-26-methoxyspirost-5-en-3 *β*-ol 3-O-*α*-L-rhamnopyranosyl-(1→2)-O-[*α*-L-arabinopyranosyl-(1→3)]-*β*-D-glucopyranoside	C_45_H_72_O_17_	MeOH	Bulbs	*L. dauricum*	Mimaki et al. (1992)
54	(25*R*,26*R*)-26-methoxyspirost-5-en-3 *β*-ol 3-O-*α*-L-rhamnopyranosyl-(1→2)-O-[*β*-D-glucopyranosyl-(1→4)]- *β*-D-glucopyranoside	C_46_H_74_O_18_	MeOH	Bulbs	*L. dauricum*	Mimaki et al. (1992)
55	(25*R*)-spirost-5-en-3 *β*-ol (diosgenin) 3-O-*α*-L-rhamnopyranosyl-(1→2)-O-[*α*-L-arabinopyranosyl-(1→3)]-*β*-D-glucopyranoside	C_45_H_72_O_17_	MeOH	Bulbs	*L. dauricum*	Mimaki et al. (1992)
56	(25*R*)-3 *β*,17 *α*-dihydroxy-5 *α*-spirostan-6-one 3-O-*α*-L-rhamnopyranosyl-(1→2)-*β*-D-glucopyranoside	C_39_H_62_O_14_	MeOH	Bulbs	*L. dauricum*	Mimaki et al. (1992)
57	(25*R*)-3 *β*, 17 *α*-dihydroxy-5 *α*-spirostan-6-one 3-O-*α*-L-rhamnopyranosyl-(1→2)-O-[*α*-L- arabinopyranosyl-(1→3)]-*β*-D-glucopyranoside	C_39_H_71_O_19_	MeOH	Bulbs	*L. dauricum*	Mimaki et al. (1992)
58	(20*R*,22*R*)-3 *β*,20,22-trihydroxy-5 *α*-cholestan-6-one (tenuifoliol) 3-O-*α*-L-rhamnopyranosyl-(1→2)-*β*-D-glucopyranoside	C_39_H_66_O_14_	MeOH	Bulbs	*L. dauricum*	Mimaki et al. (1992)
59	Lililancifoloside A	C_44_H_69_H_11_	EtOH	Bulbs	*L. lancifolium*	Yang et al. (2012)
60	Diosgenin	C_27_H_42_O_3_	EtOH	Bulbs	*L. Brownii*	Ji and Ding (2003)
61	Tigogenin	C_27_H_44_O_3_	EtOH	Bulbs	*L. Brownii*	Ji and Ding (2003)
62	(22*R*,25*R*)-spirosol-5-en-3*β*-yl O-*α*-l-rhamnopyranosyl-(1->2)-[6-O-acetyl-*β*-D-glucopyranosyl-(1->4)]-*β*-D-glucopyranoside	C_47_H_76_O_17_	EtOH	Bulbs	*L. longiflorum*	Munafo et al. (2010)
63	(25*R*)-26-O-(*β*-D-glucopyranosyl)-furost-5-en-3*β*,22*α*,26-triol 3-O-*α*-l-rhamnopyranosyl-(1->2)-*α*-l-arabinopyranosyl-(1->3)-*β*-D-glucopyranoside	C_50_H_82_O_22_	EtOH	Bulbs	*L. longiflorum*	Munafo et al. (2010)
64	(25*R*)-26-O-(*β*-D-glucopyranosyl)-furost-5-en-3*β*,22*α*,26-triol 3-O-*α*-l-rhamnopyranosyl-(1->2)-*α*-l-xylopyranosyl-(1->3)-*β*-D-glucopyranoside	C_51_H_85_O_22_	EtOH	Bulbs	*L. longiflorum*	Munafo et al. (2010)
65	(25*R*)-26-[(*β*-D-glucopyranosyl)oxy]-22*α*-hydroxyfurost-5-en-3*β*-yl O-*α*-l-arabinopyranosyl-(1→3)-O-[*β*-D-glucopyranosyl-(1→4)]-O-[*α*-l-rhamnopyranosyl-(1→2)]-*β*-D-glucopyranoside	C_56_H_92_O_27_	MeOH	Bulbs	*L. pumilum*	Matsuo et al. (2015)
66	(25*R*)-26-[(*β*-D-glucopyranosyl)oxy]-22*α*-hydroxyfurost-5-en-3*β*-yl O-*α*-l-arabinopyranosyl-(1→4)-O-[*β*-D-glucopyranosyl-(1→3)]-O-[*α*-l-rhamnopyranosyl-(1→2)]-*β*-D-glucopyranoside	C_56_H_92_O_27_	MeOH	Bulbs	*L. pumilum*	Matsuo et al. (2015)
67	(25*R*)-26-[(*β*-D-glucopyranosyl)oxy]-furosta-5,20 (22)-dien-3*β*-yl *O*-*α*-l-arabinopyranosyl-(1→3)-*O*-[*β*-D-glucopyranosyl-(1→4)]-*O*-[*α*-l-rhamnopyranosyl-(1→2)]-*β*-D-glucopyranoside	C_56_H_90_O_26_	MeOH	Bulbs	*L. pumilum*	Matsuo et al. (2015)
68	(25*R*)-26-[(*β*-D-glucopyranosyl)oxy]-furosta-5,20 (22)-dien-3*β*-yl *O*-*α*-l-arabinopyranosyl-(1→4)-*O*-[*β*-D-glucopyranosyl-(1→3)]-*O*-[*α*-l-rhamnopyranosyl-(1→2)]-*β*-D-glucopyranoside	C_56_H_90_O_26_	MeOH	Bulbs	*L. pumilum*	Matsuo et al. (2015)
Phenolics						
69	1-O-feruloylglycerol	C_13_H_16_O_6_	EtOH	Bulbs	*L. lancifolium*	Luo et al. (2012)
70	1-O-p-coumaroyl-glycerol	C_12_H_14_O_5_	EtOH	Bulbs	*L. lancifolium*	Luo et al. (2012)
71	1-O-caffeoyl-3-O-p-coumaroylglycerol	C_21_H_20_O_8_	EtOH	Bulbs	*L. lancifolium*	Luo et al. (2012)
72	1,2-O-diferuloylglycerol	C_23_H_24_O_9_	EtOH	Bulbs	*L. lancifolium*	Luo et al. (2012)
73	1,3-O-diferuloylglyc-erol	C_23_H_24_O_9_	EtOH	Bulbs	*L. lancifolium*	Luo et al. (2012)
74	1-O-feruloyl-3-O-p-coumaroylglycerol	C_22_H_22_O_8_	EtOH	Bulbs	*L. lancifolium*	Luo et al. (2012)
75	1,3-O-di-p-coumaroylglycerol	C_21_H_20_O_7_	EtOH	Bulbs	*L. lancifolium*	Luo et al. (2012)
76	Carvacrol-2-O-*β*-D-apiofuranosyl-(1→6)-*β*-D-glucopyranoside	C_21_H_32_O_10_	EtOH	Root	*L. dauricum*	Xia et al. (2022)
77	1-methyl-3-isopropylphenol-4-O-*β*-D-apiofuranosyl-(1→6)-*β*-D-glucopyranoside	C_21_H_32_O_10_	EtOH	Root	*L. dauricum*	Xia et al. (2022)
78	P-methoxythymol-5-O-*β*-D-apiofuranosyl-(1→6)-*β*-D-glucopyranoside	C_22_H_34_O_11_	EtOH	Root	*L. dauricum*	Xia et al. (2022)
79	8-O-4′ neolignan enantiomers (5a/5b)	C_21_H_26_O_7_	EtOH	Root	*L. dauricum*	Xia et al. (2022)
80	1-O-feruloyl-2-O-p-coumaroylglycerol	C_22_H_22_O_8_	MeOH	Bulbs	*L. brownii*	Ma et al. (2017)
81	1,3-O-diferuloylglycerol	C_23_H_24_O_9_	MeOH	Bulbs	*L. brownii*	Ma et al. (2017)
82	P-coumaric acid	C_6_H_4_O_4_	MeOH	Bulbs	*Lilium* spp.	Wang et al. (2015)
83	Ferulic acid	C_10_H_10_O_4_	MeOH	Bulbs	*Lilium* spp.	Wang et al. (2015)
84	Chlorogenic acid	C_16_H_18_O_9_	MeOH	Bulbs	*Lilium* spp.	Wang et al. (2015)
85	1-O-p-coumaroyl glycerol	C_13_H_14_O_4_	EtOH	Bulbs	*L. lancifolium*	Zhao et al. (2019)
86	1-O-caffeoyl-3-O-p-coumaroyl glycerol	C_21_H_20_O_8_	EtOH	Bulbs	*L. lancifolium*	Zhao et al. (2019)
87	1-O-p-coumaroyl-3-O-caffeoyl-3-hydroxy glycerol	C_21_H_20_O_9_	EtOH	Bulbs	*L. lancifolium*	Zhao et al. (2019)
88	1-O-caffeoyl-3-O-feruloyl glycerol	C_22_H_21_O_9_	EtOH	Bulbs	*L. lancifolium*	Zhao et al. (2019)
89	1-O-caffeoyl-3-O-sinapoyl glycerol	C_23_H_22_O_10_	EtOH	Bulbs	*L. lancifolium*	Zhao et al. (2019)
90	P-coumaroyl	C_9_H_8_O_3_	EtOH	Bulbs	*L. lancifolium*	Zhao et al. (2019)
91	1,3-O-p-coumaroylacetyl glycerol	C_23_H_22_O_9_	EtOH	Bulbs	*L. lancifolium*	Zhao et al. (2019)
Phenylpropanoids						
s92	3-O-acetyl-1-O-caffeoylglycer-ol	C_14_O_7_H_16_	EtOH	Bulbs	*L. brownii*	Hui, C. et al. (2024a)
93	3-O-acetyl-1-O-p-coumaroylglycerol	C_14_O_6_H_16_	EtOH	Bulbs	*L. brownii*	Hui, C. et al. (2024a)
94	Regaloside A	C_18_H_24_O_10_	EtOH	Bulbs	*L. brownii*	Seo et al. (2024)
95	Regaloside B	C_20_H_26_O_11_	EtOH	Bulbs	*L. brownii*	Seo et al. (2024)
96	Regaloside C	C_18_H_24_O_11_	EtOH	Bulbs	*L. brownii*	Seo et al. (2024)
97	Regaloside E	C_20_H_26_O_12_	EtOH	Bulbs	*L. brownii*	Seo et al. (2024)
98	Regaloside F	C_19_H_26_O_11_	EtOH	Bulbs	*L. brownii*	Seo et al. (2024)
99	Regaloside H	C_18_H_24_O_10_	EtOH	Bulbs	*L. brownii*	Seo et al. (2024)
100	Regaloside I	C_20_H_26_O_11_	EtOH	Bulbs	*L. brownii*	Seo et al. (2024)
101	Regaloside K	C_18_H_24_O_11_	EtOH	Bulbs	*L. brownii*	Seo et al. (2024)
102	(2,4,6-trichloro-3-hydroxy-5-methoxyphenyl)methyl *β*-D-glucopyranoside	C_14_H_17_Cl_3_O_8_	EtOH	Bulbs	*L. regale*	Yan et al. (2021)
103	(2,4-dichloro-3,5-dimethoxyphenyl)methyl 6-O-*β*-D-glucopyranosyl-*β*-D-glucopyranoside	C_21_H_30_Cl_2_O_13_	EtOH	Bulbs	*L. regale*	Yan et al. (2021)
104	4-chloro-3-methoxy-5-methylphenyl 6-O-(6-deoxy-*β*-L-mannopyranosyl)-*β*-D-glucopyranoside	C_20_H_29_ClO_11_	EtOH	Bulbs	*L. regale*	Yan et al. (2021)
Others						
105	*Lilium*tide A	C_21_H_29_N_3_O_6_	MeCO	Bulbs	*L. davidii* var. *unicolor*	Zhang H. et al. (2022)
106	*Lilium*tide B	C_27_H_39_N_3_O_11_	MeCO	Bulbs	*L. davidii* var. *unicolor*	Zhang H. et al. (2022)

EtOH, ethanol; MeOH, methanol; MeCO, acetone.

**TABLE 2:** Monosaccharides composition, molecular weight, structures, and bioactivities of polysaccharides purified from *Lilium* spp.

No.	Polysaccharides	Monosaccharide composition	*M.W.* (Da)	Structures	Bioactivities	Refs
1	LDP	D-mannose, D-glucose, D-galactose, with molar ratios of 10 : 19: 1	5.17 × 10^4^	The main chain of the LDP is primarily made up of a 1,4-linked form for -Glc and a 1,3-linked form for -Man, with approximate molecule ratios 2 : 1. On average, there is one 1,6-linked form for -Gal or one 1,3-linked form for -Man residues which can be substituted at 6-O from among 30 sugar residues	ND	Zhang et al. (2010)
2	LLPS-1	Glucose: mannose = 2:1; with a trace amount of arabinose and galactose	3.505 × 10^5^	ND	Immune enhancement activity	Chen et al. (2014)
3	LLPS-2	Glucose: mannose = 1:1; with a minuscule amount of arabinose	4.033 × 10^5^	ND	Immune enhancement activity	Chen et al. (2014)
4	LLPS-3	Arabinose: galactose: glucose: mannose = 2:2:2:1	1.462 × 10^5^	ND	Immune enhancement activity	Chen et al. (2014)
5	LP2-1	L-rhamnopyranose, D-arabinofuranose, D-glucopyranose and D-galactopyranose in the molar ratio of 1.88:2.13:1.00:2.50	8.52 × 10^6^	Major functional groups of LP2-1 were ACOOA and AOH	Antioxidant activity	Gao et al. (2015)
6	LLP-1	Mannose, rhamnose, glucuronic acid, galacturonic acid, glucose, galactose, arabinose glucose and galactose	2.25 × 10^6^	ND	Antioxidant activity	Xu et al. (2016)
7	LLP-2	Mannose, rhamnose, glucuronic acid, galacturonic acid, glucose, galactose, arabinose glucose and galactose	2.02 × 10^6^	ND	Antioxidant activity	Xu et al. (2016)
8	LLP-3	Mannose, rhamnose, glucuronic acid, galacturonic acid, glucose, galactose, arabinose glucose and galactose	2.08 × 10^6^	ND	Antioxidant activity	Xu et al. (2016)
9	LLP-1A	Mannose and glucose at a molar ratio of 1.77:1	7.861 × 10^4^	The presence of uronic acid, pyranose rings and *β*-glycosidic bonds	Immune enhancement activity	Pan et al. (2017)
10	LDP-2	four kinds of monosaccharides (Lyxose, Mannose, Glucose, and Galactose in an approximate weight ratio of 6.74: 6.28: 76.50: 10.48)	6.2 × 10^4^	ND	Hypoglycemic activity	Wang F. et al. (2018)
11	BHP-1	Glucose and mannose in a relative molar ratio of 5.9:2.0	1.93 × 10^5^	Mainly contained *α*-(1→4)-linked D-glucopyranosyl	Antioxidant activity	Hui et al. (2019b)
12	BHP-2	Glucose, galactose, mannose and arabinose with approximate molar ratios of 8.3:1.5:1.0:1.1	3.52 × 10^4^	Mainly contained *α*-(1→4)-linked D-glucopyranosyl	Antioxidant activity	Hui et al. (2019b)
13	LPR	Glucose and mannose with a molar ratio of2.9:3.3	5.12 × 10^4^	The backbonemainly contained *β*-(1→4)-linked D-glucopyranosyl and *β*-(1→4)-linkedDmannopyanosyl, and the branches probably linked at O-2 and/or O-3 ofthe mannosyl and glucosyl residues	Antioxidant activity	Hui et al. (2019a)
14	L01-B1	Rhamnose, glucuronic acid, galacturonic acid, galactose, and arabinose in a molar ratio of 13.4: 1.8: 11.1: 37.2: 36.5	4.39 × 10^4^	The backbone was composed of 1, 6-*β*-Galp, 1, 4-*α*-GalpA, and 1, 2-*α*-Rhap, whereas the branches included 1, 5-*α*-Araf, 1, 4-*β*-Galp, and T-*β*-GlcpA attached to C-4 of rhamnose, and 1, 3-*β*-Galp and T-*β*-Galp linked to C-3 of galactose	ND	Li M. et al. (2025)
15	WLBP-A3-c	Homogalacturonan, rhamnogalacturonan I, and rhamnogalacturonan domains, with mass ratios of 76.0: 17.2:6.8	5.9 × 10^4^	Composed of repeating units of [→2)-*α*-L-Rhap-(1→4)-*α*-D-GalpA-(1→] with highly branched neutral sugar side chains at the O-4 position of Rhap	Antioxidant activity	Zhao et al. (2024)
16	LBP	Mannose and glucose of the corresponding molar ratios are 0.582 and 0.418	3.12 × 10^5^	ND	Immune enhancement activity	Li X. J. et al. (2024)
17	LLP11	Mannoglucan	1.2 × 10^4^	A backbone of→4)-*α*-D-Glcp-(1→and→4)-*β*-D-Manp-(1→with a branch of T-*α*-D-Glcp-(1→substituted at C-6 of→4,6)-*α*-D-Glcp-(1→	ND	Bai et al. (2024)

ND, not detected; *M.W.*, molecular weight.

### 4.1 Saponins

Saponins are ubiquitous in the plant kingdom, featuring a structure with a triterpene or steroid aglycone linked to one or more sugar chains. Owing to their physicochemical (surfactant) properties and diverse biological activities, including anticancer and anticholesterol effects, their applications in the food, cosmetics, and pharmaceutical industries are continuously expanding (Guclu-Ustundag and Mazza, 2007). Numerous studies have reported that lilies are rich in diverse saponin metabolites. So far, researchers have successfully isolated and chemically characterized a total of 68 saponin metabolites (**1–68)** from various medicinal parts of *Lilium* spp., such as bulbs and roots. It is widely reported that *L. lancifolium* is rich in various steroidal saponins. Researchers isolated nine previously undescribed metabolites (**1–9)** and one known metabolite (**10)** from the bulbs of *L. lancifoliumL* Thunb. Among these, metabolites **1** and **2** possess unique structures based on the known polyoxygenated pregnane skeleton. These metabolites were characterized by nuclear magnetic resonance (NMR) spectroscopy and high-resolution mass spectrometry (HRMS) (Liu, S. et al., 2025b).

Four steroidal glycosides (**11–14)** and five known ones (**15–19)** were isolated from the bulbs of *L. lancifolium*. All isolated metabolites were evaluated for cytotoxicity against MCF-7, MDA-MB-231, HepG2, and A549 tumor cell lines. Among them, metabolite 6 emerged as the most potent, exhibiting IC_50_ values of 3.31, 5.23, 1.78, and 1.49 μM, respectively. Notably, metabolites **3**, **5**, and **9** also displayed selective cytotoxic effects. Subsequent mechanistic studies revealed that metabolite 6 arrests HepG2 cells at the G_2_/M phase and induces apoptosis (Zhou J. et al., 2024). Two previously undescribed spirostanol saponins (20–21) and two new furostanol saponins (22–23) were isolated from the fresh bulbs of Lilium longiflorum together with several known saponins (Mimaki et al., 1994). Two previously undescribed steroidal saponins (**24–25)** were isolated from the fresh bulbs of *Lilium martagon* (Satou et al., 1996). Two steroidal saponins (**26–27)** from *Lilium candidumL*. and their structures were derived mainly from NMR and mass spectra (Haladová et al., 1998). Four new metabolites were isolated from the fresh bulbs of *Lilium candidum* (Mimaki et al., 1998). Five new spirostanol saponins and a previously undescribed furostanol saponin (**32–37)** were isolated from the fresh bulbs of *Lilium candidum* (Mimaki et al., 1999). metabolites **38** and **39** are isomeric structures of 3-L-rhamnopyranosyl-12D-glucopyranosyloxyspirost-5-en-27-ol, which were isolated from *Lilium candidum* (Haladova et al., 2011). metabolites **42** and **43** are novel steroidal saponins isolated from the fresh bulbs of *L. brownii* (Mimaki and Sashida, 1990). The *bulbs* of *L. brownii* led to the isolation of seven steroidal saponins (**44–50**) (Yang et al., 2012). Two new steroidal saponins (**51–52**) were isolated from the fresh bulbs of *Lilium regale* (Mimaki et al., 1993). The bulbs of *Lilium dauricum* yielded six isolated steroidal glycosides (Mimaki et al., 1992). Lililancifoloside A (**59**) is a previously undescribed steroidal saponin (Yang et al., 2012). Diosgenin (**60**) and tigogenin (**61**) is a steroidal saponin that has been isolated from *L. brownii* (Ji and Ding, 2003).A steroidal glycoalkaloid (**62**) and two furostanol saponins (**63–64**) were isolated from the bulbs of *L. longiflorum* (Munafo et al., 2010). Four novel steroidal glycosides (**65–68**), containing a 2,3,4-trisubstituted *β*-D-glucopyranosyl group, have been successfully isolated from the bulbs of *Lilium pumilum* (Matsuo et al., 2015).

### 4.2 Phenolics

Studies indicate that dietary phenolic acids, monophenols, and polyphenols can inhibit the invasive and metastatic behaviors of various cancer cells (such as adhesion, migration, and angiogenesis) both *in vitro* and *in vivo*. Therefore, daily consumption of natural foods rich in phenolics may help prevent cancer metastasis (Djafarou et al., 2025). To date, researchers have isolated 23 phenolic metabolites from lily bulbs (**69–91)**. Phenolic metabolites are very important active metabolites in *L. lancifolium*, which play a significant role in antioxidation, antitumor (Sharifi-Rad et al., 2023), and treatment of cardiovascular diseases (Sharifi-Rad et al., 2021), while phenylpropanoid glycerol esters are common phenolic metabolites in the plant kingdom. In this study, preparative separation of seven phenylpropenoid glycerides (**69–75)** from the bulbs of *L. lancifolium*, was conducted by high-speed counter-current chromatography (HSCCC) with a two-phase solvent system composed of n-hexane-ethyl acetate-methanol-0.05% aqueous trifluoroacetic acid (TFA) (3:5:3:5, v/v/v/v). Their structures were identified by ESI-MS, 1H NMR and 13C NMR spectra (Luo et al., 2012).

Three phenolic glycosides and a pair of 8-*O*-4′ neolignan enantiomers (5A:/5B) (**76–79**), were isolated from the roots of *Lilium dauricum* (Xia et al., 2022). In the study, Ma et al. (2017)using bioassay-guided isolation method, two phenylpropenoid acylglycerols, 1-O-feruloyl-2-O-p-coumaroylglycerol (**80**) and 1,3-O-diferuloylglycerol (**81**), were obtained and identified from the chloroform fraction of the bulbs of *L. brownii* var. viridulum (Ma et al., 2017). metabolite **82**, **83**, and **84** is a phenolic metabolite that occurs in *Lilium* spp. (Wang et al., 2015). metabolite **85–91** is a phenolic metabolite that was isolated from *L. lancifoliumL* (Zhao et al., 2019).

### 4.3 Phenylpropanoids

A variety of fruits, vegetables, cereals, beverages, spices, and botanical drugs contain phenylpropanoids and their derivatives, which are plant secondary metabolites. They have diverse roles, such as antibacterial, antioxidant, anti-inflammatory, anti-diabetic, and anti-cancer activities, along with nephroprotective, neuroprotective, cardioprotective, and hepatoprotective effects (Neelam et al., 2019). So far, 13 phenylpropanoid metabolites (**92–104)** have been successfully isolated and chemically identified from lily bulbs and roots. Based on their substructure types, these metabolites can be classified into simple phenylpropanoids, lignans, and coumarins. In the study, two phenylpropanoid metabolites including 3-O-acetyl-1-O-caffeoylglycerol (**92**) and 3-O-acetyl-1-O-p-coumaroylglycerol (**93**) were isolated from the bulbs of *L. brownii*. Their structures were identified by spectroscopic method and the effect on monoamine oxidase activity was determined using an enzyme labeling method. The results show **92** and **93** have anti-monoamine oxidase activity with 20.96% and 22.31% inhibition rates at 50 μg/mL, respectively (Hui, C. et al., 2024a). A simultaneous quantitative method was developed for the quality control of Bulbs of *L. lancifoliumL* Thunb. (BLL) using high-performance liquid chromatography coupled with a photodiode array detector (HPLC-PDA), and their antioxidant effects were evaluated. Eight regalosides (i.e., regaloside A, B, C, E, F, H, I, and K) (**94–101)** were selected as marker substances. The method was validated with respect to linearity, sensitivities (limit of detection (LOD) and limit of quantitation (LOQ)), accuracy, and precision (Seo et al., 2024).

Three previously undescribed chlorophenyl glycosides, (2,4,6-trichloro-3-hydroxy-5-methoxyphenyl)methyl *β*-D-glucopyranoside (**102**), (2,4-dichloro-3,5-dimethoxyphenyl)methyl 6-O-*β*-D-glucopyranosyl-*β*-D-glucopyranoside (**103**) and 4-chloro-3-methoxy-5-methylphenyl 6-O- (6-deoxy-*β*-L-mannopyranosyl)-*β*-D-glucopyranoside (**104**) were obtained from *L. regale*. The absolute configurations of these new finds were elucidated by comprehensive analyses of spectroscopic data combined with acid hydrolysis derivatization. (2,4-dichloro-3,5-dimethoxyphenyl) methyl 6-O-*β*-D-glucopyranosyl-*β*-D-glucopyranoside (**103**) can inhibit the proliferation of lung carcinoma A549 cells with an IC_50_ value of 29 μΜ (Yan et al., 2021).

### 4.4 Other metabolites

Up to date, apart from the chemical metabolites listed above, only few metabolites have been also investigated. Briefly, twenty-five water-soluble metabolites were isolated from the bulbs of *L. davidii* var. unicolor, including two metabolites termed *Lilium*tides A and B (**105–106**) by Zhang H. et al. (2022). To confirm further the absolute configuration of *Lilium*tide A (**105**), and accumulate enough sample to study the anti-insomnia effect, a total synthesis was achieved by four steps (Zhang H. et al., 2022).

Polysaccharides, as important biomacromolecules, exhibit favorable biological properties, interact with various cell membrane structures, participate in diverse pharmacological activities, and are widely distributed throughout biological systems (Chen L. et al., 2025). Polysaccharides are among the most extensively studied classes of metabolites, exhibiting significant pharmacological effects in *Lilium* spp., from which 17 polysaccharide metabolites have been successfully isolated and purified. It is reported that they exhibit various pharmacological activities, including antioxidant, immunomodulatory, and hypoglycemic effects.The polysaccharide LDP, derived from *Lilium* spp., is an amorphous powder composed of three monosaccharides: D-mannose (D-Man), D-glucose (D-Glc), and D-galactose (D-Gal), in approximate molar ratios of 10:19:1 (Zhang et al., 2010). In 2014, Chen et al. (2014) extracted water-soluble polysaccharides from tiger lily using an ultrasound-assisted method, subsequently separating and purifying LLPS-1, LLPS-2, and LLPS-3, with molecular weights of 350.5, 403.3, and 146.2 kDa, respectively. LLPS-1 and LLPS-2 primarily consisted of glucose and mannose in molar ratios of nearly 1:2 and 1:1, respectively. In contrast, LLPS-3 was primarily composed of arabinose, galactose, glucose, and mannose in a molar ratio of nearly 2:2:2:1 (Chen et al., 2014). A novel polysaccharide fraction (LP2-1) was isolated and purified from the edible bulbs of *Lilium* lancifolium Thunb. LP2-1 had an average molecular weight of approximately 8.52 × 10^3^ kDa and was mainly composed of L-rhamnopyranose, D-arabinofuranose, D-glucopyranose and D-galactopyranose in the molar ratio of 1.88:2.13:1.00:2.50 (Gao et al., 2015). In other study, LLP-1, LLP-2, and LLP-3 three novel polysaccharide fractions were purified from the leaves of *Lilium* lancifolium (Xu et al., 2016).

In summary, the monosaccharide composition, molecular weight, structural characteristics, and biological activities of the aforementioned purified polysaccharides from *Lilium* spp. are presented in Table 2. The polysaccharides of *Lilium* spp., as detailed in Table 2, feature a composition of key monosaccharides including mannose, glucose, galactose, arabinose, rhamnose, glucuronic acid, galacturonic acid, and lyxose. Consequently, polysaccharides derived from *Lilium* spp. demonstrate diverse applications in the medical and healthcare fields, and their potential uses in scientific research and clinical settings are actively being explored.

In summary, 123 chemical metabolites have been isolated and identified from *Lilium* plants to date, with the core metabolites including saponins (68 species, such as metabolites 6, 9, 103, and total tigogenin saponins), phenolics (23 species, such as phenylpropanoid glycerol esters 69-75 and 1-O-Resina Ferulaen-2-O-p-coumaroyl glycerol), phenylpropanes (13 species, covering regalin A-K, metabolites 92-93, and chlorophenyl glycosides 102–104), and structural polysaccharides (17 species, including LDP, LLPS-1/2/3, LP2-1, and LP60-1, etc.). The mechanisms of action of some chemical metabolites have been elucidated, metabolite 6 exerts anti-neoplasms activity by arresting HepG2 Cell in the G_2_/M phase and inducing Apoptosis; regalin A achieves anti-Depression effect via regulating the PI3K/AKT/mTOR signaling pathway; structural polysaccharides achieve hypoglycemic and immunisation effects by inhibiting alglucosidase alfa/*α*-Diastase and interfering with the TLR4/NF-κB pathway, respectively; phenylpropanoid glycerol esters exhibit antioxidant effects by scavenging free radicals and chelating Metal ions. However, the structure-activity relationship of active metabolites in *Lilium* has not been systematically revealed and still needs further investigation.

## 5 Pharmacological properties

**FIGURE 3:**
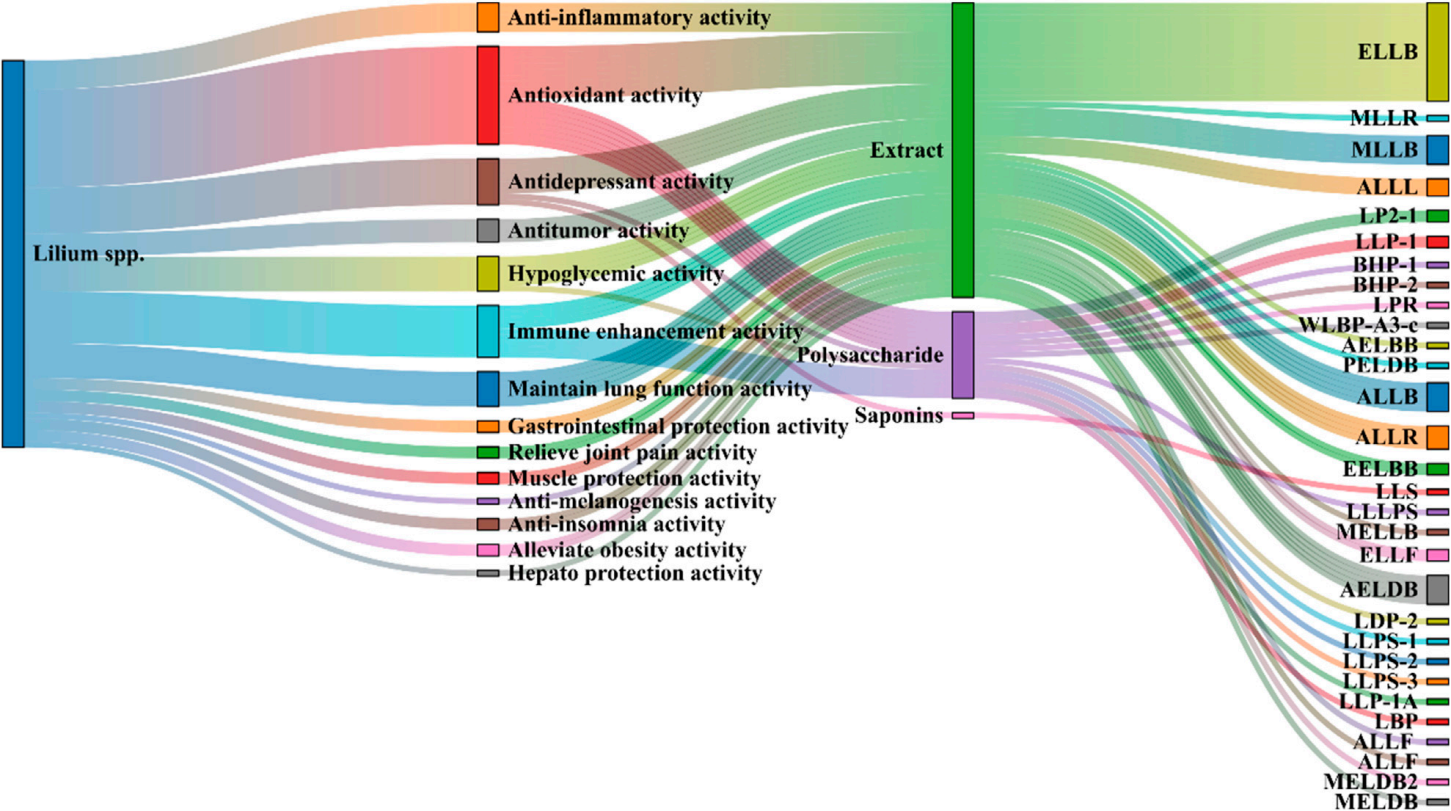
Sankey diagram of pharmacological activities of the *Lilium spp.*

**FIGURE 4:**
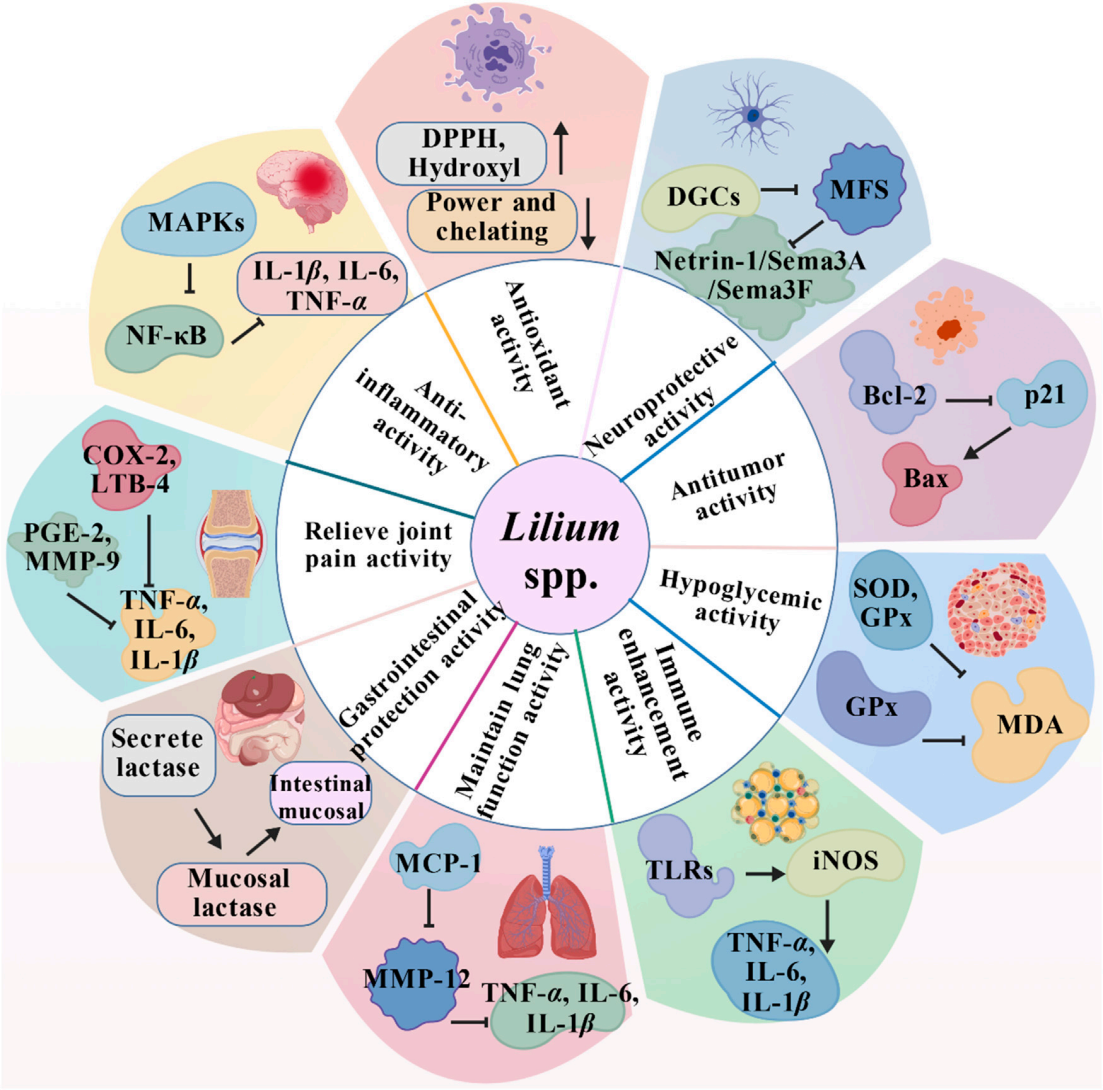
Main pharmacological activities of the *Lilium* spp.

As previously mentioned, lilies have been widely used in traditional medicine in many countries for thousands of years to treat and prevent various diseases. Currently, numerous studies have reported on the pharmacological activities of *Lilium* spp. extracts from most of its effective chemical metabolites exhibit a wide range of pharmacological activities, including hypoglycemic, anti-inflammatory, antioxidant, antidepressant, antitumor, neuroprotective, immune-enhancing, lung function maintenance, intestinal protection, and anti-insomnia effects. Consequently, *Lilium* spp. has recently garnered increasing attention. These pharmacological studies will be elaborated on in the following paragraphs. These biological activities are discussed individually in the following paragraphs, and a summary is presented in Table 3. Next, all these pharmacological findings will be summarized and discussed in the sections that follow. To this end, we listed the main pharmacological activities of the *Lilium* spp. using Sankey diagram in Figure 3, and graphically illustrated the pharmacological activities of the *Lilium* spp. in Figure 4.

**TABLE 3:** Pharmacological effects of crude extracts and bioactive metabolites of *Lilium* spp.

No.	Metabolites/Extracts	Types	Testing subjects	Doses/Duration	Effects/Mechanisms	Positive controls/Negative control	Refs
Anti-inflammatory activity							
1	ELLB	*In vitro*	RAW 264.7 cells	50–200 μg/mL for 24 h	IL-1β, IL-6, and TNF-α levels ↓; p-PI3K, p-Akt, and p-IKK protein expression ↓; MyD88 and TRAF6 expression levels ↓; IFN-*β*, secreted by activated IRF3 ↓	Indomethacin/Lipopolysaccharide	Sim et al. (2020)
2	MLLR	*In vitro*	RAW 264.7 cells	10, 20, 50, and 100 g/mL for 24 h	NO, PGE2, IL-6 and TNF-α production ↓; ERK1/2 and JNK and translocation of the NF-κB p65 subunit into nuclei ↓; interleukin-4 and interleukin-13 ↓	NM/Lipopolysaccharide	Kwon et al. (2010)
3	ELLB	*In vitro*	RAW 264.7 cells	0–300 μg/mL for 24 h	NO, iNOS, COX2, and TNF-α levels ↓; MyD88- and TRIF-induced NF-κB transcriptional activation and the nuclear translocation of NF-κB transcription factors, the NF-κB signaling pathway ↓	Lipopolysaccharide/no Lipopolysaccharide	Han et al. (2018)
4	MLLB	*In vitro*	RAW 264.7 cells	5–100 μg/mL for 24 h	IC_50_ were 9.12 µM and 12.01 µM; PGE2, IL-1β, IL-6, and TNF-α levels ↓; NOS, COX-2 protein levels ↓; NF-κB p65 subunit, MAPKs pathway ↓	Dexamethasone/Lipopolysaccharide	Ma et al. (2017)
5	ALLL	*In vitro*	LPS-induce HaCaT cells	25, 50, 100, 200, 400, 800 μg/ML for 24 h	IL-6, iNOS level ↓	Aspirin/Lipopolysaccharide	Hu et al. (2025)
Antioxidant activity							
6	ELLB	*In vitro*	CCK-8 assay (assess PC12 cells)	50 μM for 24 h	Antioxidant activity ↓	N-Acetylcysteine/RPMI-1640 Medium	Liu et al. (2025a)
7	ELLB	*In vitro*	DPPH assays	2.5, 5, and 10 mg/mL	FRAP activity ↑	Lipopolysaccharide + Indomethacin/Lipopolysaccharide	Sim et al. (2020)
8	ALLL	*In vitro*	DPPH, Hydroxyl, Superoxide, and Fe^2+^ chelating assays	0.125, 0.25, 0.5, 1.0, 2.0, 3.0, 4.0, and 5.0 mg/mL	DPPH assays EC_50_ were 5.54, 2.34, and 1.43 mg/mL; Hydroxyl assays EC_50_ were 0.71, 0.52, 0.53, and 0.22 mg/mL; Superoxide assays EC_50_ were 0.09, 0.07, and 0.06 mg/mL; Fe^2+^ chelating assays EC_50_ were 0.71, 0.70, and 0.60 mg/mL	Ethylenediaminetetraacetic Acid/Deionizedwater	Xu et al. (2016)
9	ELLB	*In vitro*	DPPH, Hydroxyl, reducing power, and Fe^2+^ chelating assays	0.25, 0.5, 0.75, 1.0, 2.0, 3.0, 4.0 and 5.0 mg/mL	DPPH and hydroxyl radicals scavenging activities ↑; power and chelating activity on ferrous ion ↓	Ethylenediaminetetraacetic Acid/Deionizedwater	Gao et al. (2015)
10	MLLB	*In vitro*	DPPH, ABTS, cupric-reducing, and hydroxyl assays	NM	Antioxidant activity ↑	Trolox/Deionizedwater	Jin et al. (2012)
11	ELLB	*In vitro*	DPPH, ABTS, FRAP assays	100 μL	IC_50_ = 0.033 µM, Antioxidant capability; radical cation scav-enging capacities; Ferric reducing antioxidant power ↑	Trolox/Deionizedwater	Luo et al. (2012)
12	ELLB	*In vitro*	DPPH, ABTS, and FRAP assays	NM	Antioxidant activity↑	Trolox/Deionizedwater	Liang et al. (2022)
13	AELBB	*In vitro*	DPPH, ABTS, and Hydroxyl radical scavenging assays; HepG2 cells	0.5, 1.0, 2.0, 5.0 and 10.0 mg/mL; 100 or 200 μg/mL for 24 h	IC_50_ values for DPPH, hydroxyl and ABTS radicals were 35.3, 7.1 and 36.4 mg/mL, respectively	GlcA/Deionizedwater	Zhao et al. (2024)
14	PELDB	*In vitro*	DPPH assays	0.0, 0.2, 0.4, 0.6, 0.8, 1.0 mg/mL	IC_50_ values: 0.63, 0.32, and 0.30 mg/mL, respectively	Floralfragranceextract/DealcoholizedWine	Gao et al. (2022)
Antidepressant activity							
15	ALLB	*In vivo*	Male C57BL/6 J mice with PTZ-induced seizures	500 mg/kg; p.o., daily for 14 days	Hyperactivation and ectopic migration of DGCs ↓; PTZ kindling-induced MFS ↓; MFS by regulating hippocampal Netrin-1/Sema3A/Sema3F ↓	Valproicacid/PTZ	Park and Cai (2024)
16	ELLB	*In vitro*	SH-SY5Y cell injury model induced by CORT	5, 15, 25, 50, 75, and 100 μM for 24 or 48 h	Brain-derived neurotrophic factor, tyrosine kinase receptor B phosphorylation levels ↑; phatidylinositol 3 kinase, protein kinase B, and mammalian target of rapamycin proteins expression ↑; Damage of corticosterone in SH-SY5Y cells ↓	Corticosterone/DMEMmedium	Yuan et al. (2021)
17	ALLL	*In vitro*	CORT-induced in PC12 cells	100, 150, 200, 250, and 300 μM for 24 h	UPRs (GRP78 and CHOP), Ca^2+^ level ↓; Neuronal apoptosis-related proteins expression ↓; CORT-induced DNA fragmentation ↓; Intracellular ROS levels ↓	Corticosterone/Dimethylsulfoxide	Lee et al. (2025)
18	ALLR	*In vivo*	OVX mice	1.8 g/kg, i.g., daily for 3 weeks	Hippocampal NGF, prefrontal GDNF ↑; uterine and brain regional ER*β* expression levels ↑	Ovariectomized/vehicle	Zhou et al. (2019a)
19	ALLR	*In vivo*	OVX mice	50, 100, and 200 mg/kg, i.g., daily for 6 days	The glutamate levels and NMDAR1 expression↓; the p-CaMKII/CaMKII ratio ↑	Ovariectomized/vehicle	Zhou et al. (2019b)
20	EELBB	*In vivo*	Mice with PD induced by 1-methyl-4-phenyl-1,2,3,6-tetrahydropyridine hydrochloride	Ethanol groups (1.25 g/kg, 2.5 g/kg) aqueous groups (0.78125 g/kg, 1.5625 g/kg), i. g., daily for 30 days	The number of neurons in the substantia nigra region ↑; MDA, Fe^2+^, SOD, and GSH-Px levels ↑	1-methyl-4-phenyl-1,2,3,6-tetrahydropyridinehydrochloride/Normalsaline	Hui, C. et al. (2024b)
21	LLS	*In vivo*	Mice exposure to the chronic unpredictable mild stress	50 mg/kg, i.g., daily for 30 days	COX-2, PGE2, and IL-22 protein expression ↓	Chronicunpredictablemildstress/NM	Ma et al. (2024)
22	LLLPS	*In vitro*	BV-2 cells	1 mg/L for 24 h	BV-2 microglial cell activation, inflammation, and neuron apoptosis↓	Chronicunpredictablemildstress/NM	Ma et al. (2024)
Antitumor activity							
23	ELLB	*In vitro*	Cytotoxic potential against the MCF-7, MDA-MB-231, HepG2, and A549 cell	100, 10, 1, 0.1 μM for 24 h	MCF-7, MDA-MB-231, HepG2, and A549 cell lines IC_50_ values: 3.31, 5.23, 1.78, and 1.49 μM, respectively	Paclitaxel/DimethylSulfoxide	Zhou J. et al. (2024)
24	ELLB	*In vitro*	SGC-7901 and HGC-27 cells	0, 25, 50, 100, 200, 400 μg/mL for 24 h	PCNA, anti-apoptotic protein Bcl-2 expression level ↓; p21 level, pro-apoptotic protein Bax expression ↑; Cell migration and invasion, MMP-2 expression ↓; TIMP-1 expression ↑	Cisplatin/0.3%ethanol	Zhang et al. (2020)
25	ALLB	*In vitro*	MDA-MB-231, A549, HepG2, and MCF7 cell	50–400 μg/mL	MDA-MB-231, A549, HepG2, MCF7. IC_50_ were 267.7, 131.8, 123.8, and 99.63 μg/mL, respectively	Paclitaxel/0.1% (v/v)DimethylSulfoxide	Zhang M. et al. (2022)
26	MELLB	*In vitro*	G292, A431 and KB cancer and HGF-1 normal cells	50 μg/mL	BID/MAPK14 expression ↑; decreased MDM2/BCL2/MYC expression, p53 protein-induced apoptosis↓	Doxorubicin/0.1% (w/v)DimethylSulfoxide	Partovi et al. (2023)
Hypoglycemic activity							
27	ELLF	*In vitro*	The glucose uptake and consumption of metabolites on Caco-2 cells	400, 200, 100, 50, 25, 12.5, and 6.25 μM for 48 h	Glucose by normal Caco-2 cells absorption ↑; SGLT1 and GLUT2 and mRNA transcription protein expression levels ↑; Glucose tracer 2-NBDG on Caco-2 cells uptake ↑	NM/Phosphate-BufferedSaline	Xu et al. (2021)
28	ELLB	*In vivo*	STZ induced diabetic mice	200 mg/kg, i.g., daily for 28 days	MDA in serum, liver and kidney levels ↓; SOD, CAT and GPx in the serum, liver and kidney levels ↑	Glibenclamide/DistilledWater	Zhang et al. (2014)
29	MLLB	*In vitro*	HepG2 cells, 3T3L1 adipocytes, and 3T3-L1 preadipocyte	1, 5, 10, 25, 50, 100, 200 μg/mL	Glucose consumption in HepG2 cells and 3T3-L1 adipocytes; 3T3-L1 preadipocyte differentiation ↑	Metformin/DimethylSulfoxide	Zhu et al. (2014)
30	AELDB	*In vitro*	*α*-amylase and *α*-glucosidase inhibition assay	0.125, 0.25, 0.5, 1, 2 and 4 mg/mL	IC_50_ values: 0.31 and 0.18 mg/mL, 0.27 and 0.12 mg/mL	Acarbose/Phosphate-BufferedSaline	Hui, H. et al. (2024a)
31	AELDB	*In vitro*	*α*-amylase and *α*-glucosidase inhibition assay	4, 2, 1, 0.5, 0.25 and 0.125 mg/mL	*α*-glucosidase and *α*-amylase ↓	Acarbose/Phosphate-BufferedSaline	Hui, H. et al. (2024b)
Immune enhancement activity							
32	ELLB	*In vitro*	RAW264.7 cells	0, 75, 150, 300, and 600 μg/mL for 20 h	Cytokines interleukin-6, monocyte chemotactic protein 1, tumor necrosis factor-*α* and interleukin-1*β* expression ↑; Toll-like receptor 4 and phosphorylation of the inhibitor of nuclear factor kappa-B kinase, inhibitor of NF-κB, and nuclear factor-kappa B protein expression ↑	Lipopolysaccharide/Phosphate-BufferedSaline	Pan et al. (2017)
33	ALLB	*In vitro*	RAW264.7 cells	5 μg/mL, 10 μg/mL for 24 h	TNF-α, iNOS, IL-6, IL-1β and TLRs mRNA expression ↑	Lipopolysaccharide/Dulbecco’sModifiedEagleMedium	Li X. J. et al. (2024)
34	AELDB	*In vitro*	RAW 264.7 cells	50, 25, 12.5, 6.25 and 3.125 μg/mL for 24 h	RAW 264.7 cells significantly phagocytosis, interleukin (IL)-1*β* and IL-2, acid phosphatase, and CD86 and CD80 molecular expression ↑	Lipopolysaccharide/Dulbecco’sModifiedEagleMedium	Li et al. (2021)
35	AELDB	*In vivo*	Immunosuppressive modeling mice	25, 50, and 100 mg/kg, i.g., daily for 5 days	Immune organs index, interferon-*γ*, IL-6, immunoglobulin (Ig)G and IgM, lymphocyte proliferation ↑	Cyclophosphamide/PhysiologicSaline	Li et al. (2021)
Maintain lung function activity							
36	ALLR	*In vivo*	C57BL/6 mice prior CS exposure	50 mg/kg, 100 mg/kg, and 150 mg/kg, i.g., daily for 3 weeks	Inflammatory cells (macrophages and neutrophils), pro-inflammatory cytokines (TNF-α, IL-6, IL-1β), chemokine (MCP-1), and protease (MMP-12) level ↓; Airway enlargement in CS-exposed mice ↓	Dexamethasone/DistilledWater	Lee et al. (2013)
37	ALLB	*In vivo*	COPD mouse models induced by cigarette smoke extract and porcine pancreas elastase	100 mg/kg, 200 mg/kg, i.g., daily for 22 days	Production of inflammatory mediators and infiltration of immune cells involving neutrophils and macrophages ↓; IL-6 and MIP-2 production, NF-κB ↓	Roflumilast/Phosphate-BufferedSaline	Eom et al. (2023)
38	ALLF	*In vivo*	C57BL/6J mice injected intratracheally with bleomycin	10, 30, 90 mg/kg, i.p., daily for 14 days	Fibrosis-related markers and suppressed the EMT process expression ↓	N-Acetylcysteine/DimethylSulfoxide	Liu R. et al. (2025)
39	ALLF	*In vitro*	TGF-*β*1-Induced Alteration of A549 Cell	0, 200, 400, 600, 800, 1,000 μg/mL for 48 h	*α*SMA, COL1A1 levels ↓; E-cadherin ↓; Vimentin ↑	Nintedanib/PhysiologicSaline	Liu R. et al. (2025)
40	ELLB	*In vivo*	LPS-induced pneumonia mice	100 mg/kg, 200 mg/kg, i.g., for 48 h	Inflammatory cell infiltration, proinflammatory factors (IL-6, IL-1β, TNF-α) levels ↓; (IL-6, NF-κB P65) in the lung tissue, cilia-related genes (Ttc21a, Cfap45, etc.) proteins levels ↓	Lipopolysaccharide/PhysiologicSaline	Sun et al. (2024)
41	MELDB2	*In vivo*	mice model exposed to the carbon black nanoparticles	0.1, 0.5, 2.5, 5, and 10 mg/mL i.g., for 28 days	TNF-α, IL-10, and IL-6 levels ↓	CarbonBlackNanoparticles/Phosphate-BufferedSaline	Khan et al. (2024)
Gastrointestinal protection activity							
42	ELLF	*In vitro*	GB medium pre-culture	10 mg/mL for 24 h	Bifidobacterium longum abundance ↑; *Bacteroides* thetaiotaomicron abundance ↓	Simvastatin/DistilledWater	Li Z. et al. (2025)
43	ALLB	*In vivo*	Intestinal contents and mucosa of male Kunming mice	0.15 g/mL, i.g., twice daily for 49 days	*Lactobacillus* spp. and Bifidobacteria spp growth ↑; Amylase activities in intestine contents ↑; Mucosa lactase activity ↑	Lactobacillusspp./Bifidobacteriaspp.StandardStrains/SterilizedWater	Wu et al. (2021)
Relieve joint pain activity							
44	ELLB	*In vivo*	Clinical trial (adults experiencing pain for more than 3 months with pain Visual Analog Scale scores of 30–70 mm)	1,000 mg; p.o., daily for 12 weeks	IL-6, TNF-α levels ↓; Arthritis-related pain ↓	StandardSolutions/PlaceboTablets	Jeon et al. (2024)
45	ELLB	*In vivo*	Osteoarthritis induction male beagle dogs	60 mg/kg; p.o., daily for 12 weeks	IL-6, COX-2, LTB-4, PGE-2, and MMP-9 levels ↓	GlucosamineHydrochloride/EmptyGelatinCapsules	Cho et al. (2022)
Myoprotective							
46	ELLB	*In vitro*	DEX-induced C2C12 myoblast cells	0.2 mM DEX for 24 h	C2C12 myo- tubes muscle density ↑	Resveratrol/Phosphate-BufferedSaline	Kim et al. (2024)
47	ELLB	*In vivo*	DEX-induced male C57BL/6 N the mice	100, 300 mg/kg, i.g., daily for 10 days	Myogenic protein Myod1 promoter activity ↑; Muscle protein degradation ↓	AscorbicAcid/Dulbecco’sModifiedEagleMedium	Kim et al. (2024)
Anti-melanogenesis activity							
48	ALLR	*In vitro*	B16F10 cells	0, 20, 50, 100, and 200 μg/mL for 48 h	Melanin production, core melanogenic enzymes expression ↓; Cellular tyrosinase activity as well as the mRNA and protein levels of tyrosinase, Tyrp1, and Tyrp2 ↓; Melanogenesis by effectively attenuating the CREB/Mitf signaling pathway ↓; Upstream signaling proteins, such as PKA, ERK, and p38 levels ↓	Arbutin/Dulbecco’sModifiedEagleMedium	Park et al. (2023)
Anti-insomnia activity							
49	MELDB	*In vivo*	Injected with sodium pentobarbital male SD rats	5 mg/kg, i.v., for 60 min	sleep latency ↓; sleep time ↑	Melatonin/NormalSaline	Zhang H. et al. (2022)
50	EELBB	*In vivo*	Insomnia model by intraperitoneally injection p-chlorophenylalanine	598.64 mg/kg, p.o., daily for 7 days	5-HT, MT↑; 5-HT1AR and GABA_A_R ↓; kynurenic acid, trimethylamine-N-oxide ↓	p-Chlorophenylalanine/NormalSaline	Si et al. (2022)
Alleviate obesity activity							
51	MLLB	*In vitro*	HepG2 cells	200 and 400 μg/mL for 48 h	Mitochondrial membrane potential, reactive oxygen species production, oxidative stress, and lipid accumulation ↓	AscorbicAcid/Dulbecco’sModifiedEagleMedium	Kan et al. (2021)
52	MLLB	*In vivo*	High-fat diet the mice	150, 300 mg/kg, i.g., daily for 11 weeks	Body weight gain, lipid levels in serum and liver ↓; amelliorated hepatic steatosis and hepatic-lipogenesis-related genes (SREBP-1c, FAS, ACC1, and SCD-1) expression ↑; Lipolysis genes (SRB1 and HL) and lipid oxidation genes (PPAR*α* and CPT-1) in mice fed a high-fat diet ↑	OleicAcid/NormalDiet	Kan et al. (2021)
Hepato protection activity							
53	AELDB	*In vivo*	High-fat diet induced NAFLD mice	100, 200 mg/kg i.g., daily for 4 weeks	TG, TC, HDL-C and LDL-C levels, hepatic steatosis, TNF-α and IL-1β expression, hepatic proinflammatory cytokines leves ↓	Simvastatin/NormalDiet	Li Z. et al. (2025)

NM, not mentioned; ELLB, ethanol extract of *L. lancifolium* Bulbs; MLLR, methanol extract from the *L. lancifolium* roots; MLLB, methanol extract from the *L. lancifolium* Bulbs; ELLF, ethanol extract of *L. lancifolium* flowers; ALLL, aqueous extract from the *L. lancifolium* leaves; ALLB, aqueous extract from the *L. lancifolium* Bulbus; ALLR, aqueous extract from the *L. lancifolium* roots; ALLF, aqueous extract from the *L. lancifolium* flowers; AELDB, acetone extract of *L. davidii* Bulbs; MELDB1, acetone extract of *L. davidii* Bulbs; EELBB, ethanol extract of *Lilium brownii* Bulbs; MELDB2, methanol extract of *Lilium davidii* Bulbs; MELLB, Methanol extract of *L. ledebourii* Bulbs; LLLPS, Longya *Lilium* lipopolysaccharide (LPS); LLS, Longya L. saponins; AELBB, aqueous extract of *L. brownii* Bulbs; PELDB, polyethylene glycol extract of *Lilium davidii* Bulbs; Inos, Inducuble nitric oxide synthase; COX2, cyclooxygenase-2; MAPKs, mitogen-activated protein kinases; NGF, nerve growth factor; GDNF, glial cell-derived neurotrophic factor; PCNA, proliferating cell nuclear antigen; MMP-2, matrix metalloproteinase-2; TIMP-1, Tissue inhibitor of metalloproteinases-1; TLRs, Toll-like receptors family.

### 5.1 Anti-inflammatory activity

Inflammation can contribute to various diseases, such as autoimmune, neurodegenerative diseases, and even cancer. Available anti-inflammatory drugs like aspirin and other NSAIDs may have harmful side effects despite being effective (Ruggiero et al., 2025). So, safer phytopharmaceutical anti-inflammatory metabolites are gaining attention as a better option. Anti-inflammatory activities of dried Korean and Japanese *L. lancifolium* bulb extracts were examined using RAW 264.7 cells by Sim et al. (2020). Furthermore, the anti-inflammatory effects of the ethanolic extract from Korean *L. lancifolium* bulbs were found to be mediated through both the MyD88-dependent and TRIF-dependent pathways in LPS-stimulated RAW 264.7 macrophages. In particular, pro-inflammatory protein expression through the MyD88 dependent pathway was significantly reduced upon treatment with ethanolic extracts of Korean *L. lancifolium* bulbs than those of Japanese bulbs. Taken together, Korean *L. lancifolium* bulbs could be an anti-inflammatory agent (Sim et al., 2020). Concurrently, others were also conducting similar experiments. Han et al. (2018) also investigated the anti-inflammatory activity of *Lilium* spp. bulbs and the underlying mechanism of action in macrophages using *Lilium* spp. bulb ethanol extracts (Lb-EE). The study concluded that Lb-EE inhibited the production of nitric oxide (NO) in lipopolysaccharide (LPS)-stimulated RAW264.7 cells and bone marrow-derived macrophages (BMDMs) in a Dose-dependent manner, without causing significant cytotoxicity. Furthermore, Lb-EE inhibited IKK*α*/*β*-induced activation of the NF-κB signaling pathway, and IKK inhibition significantly reduced NO production in LPS-stimulated RAW264.7 cells. In summary, these results suggest that Lb-EE plays an anti-inflammatory role by targeting IKK*α*/*β*-mediated activation of the NF-κB signaling pathway during macrophage-mediated inflammatory responses (Han et al., 2018). In addition, Kwon et al. (2010) investigated the anti-inflammatory effects of methanol extracts of the root of *L. lancifolium* (LL extracts) in LPS-stimulated Raw264.7 cells. Levels of NO, PGE2, and pro-inflammatory cytokines (IL-6 and TNF-α) in the supernatant were quantified using sandwich ELISA. Expression of COX-2 and iNOS, phosphorylation of MAPK subgroups (ERK and JNK), and NF-κB activation in cellular extracts were assessed by Western blotting and immunocytochemistry. The LL extract significantly inhibited the production of NO, PGE2, IL-6, and TNF-α in LPS-stimulated cells and suppressed the expression of iNOS and COX-2. The study concluded that the anti-inflammatory effects of *L. lancifolium* methanol extract in LPS-stimulated RAW264.7 cells may be mediated by downregulating iNOS and COX-2 by inhibiting NF-κB activation and nuclear translocation, and blocking ERK and JNK signaling (Kwon et al., 2010).

In this study, Ma et al. (2017) made use of the bioassay-guided approach to investigate the potential anti-inflammatory metabolites of LB and macrophage RAW264.7 cell line to explore the molecular mechanisms responsible for its anti-inflammatory activity. These two metabolites significantly decreased the production of nitrite oxide (NO) in lipopolysaccharide (LPS)-stimulated mouse macrophage RAW264.7 cells in a dose-dependent manner with half maximal inhibitory concentration (IC_50_) values of 9.12 µM and 12.01 µM, respectively. The study concluded that these metabolites inhibited the production of prostaglandin E2 (PGE2) and several other pro-inflammatory cytokines, such as IL-1β, IL-6, and TNF-α, and exhibited anti-inflammatory activity by acting on the NF-κB and MAPKs pathways (Ma et al., 2017). In another experiment, Hu et al. (2025) creatively established an inflammation model using HaCaT cells to assess the skin-soothing effects of the lily bulbs extract (LBE) and regaloside A. RT-qPCR was conducted to investigate the effects of LBE at 50 μg/mL, 100 μg/mL, and 200 μg/mL and regaloside A at 25 μg/mL, 50 μg/mL, and 100 μg/mL on the relative mRNA expression of IL-6 and iNOS in HaCaT cells. The study concluded that each treatment group significantly reduced the relative mRNA expression levels of IL-6 and iNOS in HaCaT cells compared to the model group. This suggests that regaloside A possesses potential anti-inflammatory activity and holds promise as a natural metabolite for developing cosmetics with skin-soothing effects (Hu et al., 2025).

**FIGURE 5:**
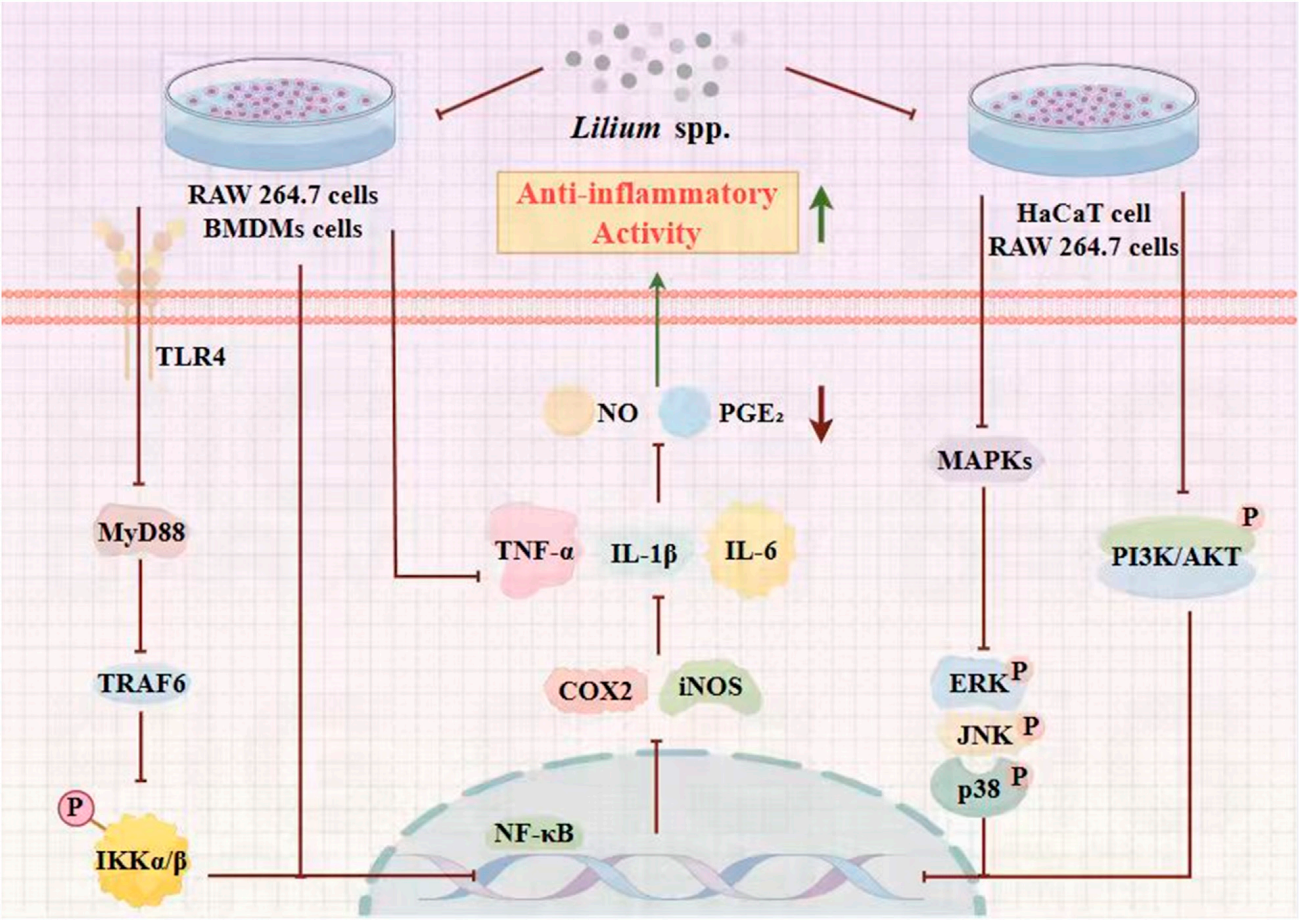
Schematic representation of underlying molecular mechanism displaying the anti-inflammatory activity of the *Lilium spp.*

In conclusion, Multiple studies have confirmed the significant anti-inflammatory activity of extracts from *Lilium* spp. These extracts primarily exert their anti-inflammatory effects by modulating various signaling pathways, including down-regulating pro-inflammatory genes such as COX-2, iNOS, IL-6, and TNF-α through blocking the classical NF-κB pathway (inhibiting IKK*α*/*β* phosphorylation, IκB degradation, and p65 nuclear translocation); concurrently suppressing the MAPK cascade (ERK, JNK) to reduce transcription factor AP-1 activation, thereby decreasing PGE_2_ and NO production; and simultaneously interfering with TLR4 adapter protein MyD88/TRIF-dependent signaling to weaken the initiation of inflammation at its source. These findings provide a scientific basis for developing *Lilium* spp. extracts as anti-inflammatory agents, suggesting their broad application prospects in the pharmaceutical and cosmetic fields. In summary, the regulatory mechanism of the *Lilium* genus on the mRNA and protein expression levels of inflammation-related factors is shown in Figure 5.

### 5.2 Antioxidant activity

Current evidence strongly indicates that oxidative stress is a key factor in various diseases, including neurodegenerative conditions such as Alzheimer’s disease, Parkinson’s disease, Down syndrome, and amyotrophic lateral sclerosis (Luan et al., 2020). Antioxidants that scavenge free radicals are highly valued for their protective properties. The health-promoting effects of antioxidants and their role in reducing disease risk are well-recognized. A wide variety of antioxidants, each with distinct functions, collectively strengthen the body’s defense mechanisms (Liu et al., 2025a). Liu et al. (2025b) isolated metabolites 1 to 10 from the bulbs of *L. lancifolium*. and investigated their cytotoxicity toward rat adrenal pheochromocytoma (PC12) cells, as well as their antioxidant effects against H_2_O_2_-induced oxidative damage in PC12 cells. The results indicated that extracts of *L. lancifolium* possess potential as antioxidants, and exhibiting varying degrees of antioxidant activity (Liu et al., 2025b). Significant antioxidant activity has also been found in lily polysaccharides. Pectic polysaccharides (WLBP-A3-c) were isolated from traditional Chinese medicines *L. brownii* by Zhao et al. (2024). Simultaneously, the antioxidant effects of diverse pectin fractions and their structural domains, produced by enzymatic hydrolysis, were assessed for their ability to scavenge different free radicals and protect H2O2-injured HepG2 cells. The study concluded that pectins rich in HG domains from *L. brownii* exhibit significant antioxidant effects. These findings reveal its potential application in the field of health products (Zhao et al., 2024). In a separate investigation, polysaccharides from Lanzhou Lily (LLPs) were successfully extracted using a polyethylene glycol-based ultrasonic-assisted enzymatic method (PEG-UAEE). The structural features of the purified LLPs were characterized by HPLC, FT-IR, and SEM, and their antioxidant potential was subsequently evaluated. The study found that LLPs have significant *in vitro* antioxidant effects, indicating their potential as natural antioxidants and food metabolites in functional foods (Gao et al., 2022).

Recently, Sim et al. (2020) aims to confirm the potential of Korean *L. lancifolium* bulbs as a functional food material by comparing the metabolites and their biological activities with Japanese *L. lancifolium* bulbs. This study analyzed the efficacy evaluations of dried Korean and Japanese *L. lancifolium* bulb extracts were performed based on the antioxidant activities. The study concluded that the content of phenolic metabolites in Korean *L. lancifolium* bulb extracts was higher than that of Japanese *L. lancifolium* bulb extracts, and the antioxidant activities of Korean *L. lancifolium* bulb extracts were superior to those of Japanese bulbs (Sim et al., 2020). Subsequently, Xu et al. (2016) extracted polysaccharides from *L. lancifolium* leaves and successfully prepared three previously undescribed polysaccharide fractions (LLP-1, LLP-2, and LLP-3). DPPH radical scavenging assay, hydroxyl radical scavenging assay, superoxide radical scavenging assay, and ferrous ion chelating assay were used. Within the concentration range of 0.125–5.0 mg/mL, the three polysaccharide fractions all exhibited significant dose-dependent scavenging effects on DPPH radicals, but their overall ability to scavenge DPPH radicals was not as strong as that of Vc. The EC_50_ values of LLP-1, LLP-2, and LLP-3 were 5.54, 2.34, and 1.43 mg/mL, respectively. Meanwhile, the chelating abilities of the three polysaccharide fractions increased with increasing concentration. At a concentration of 5.0 mg/mL, the chelating activities of LLP-1, LLP-2, and LLP-3 on Fe^2+^ were 95.21%, 93.57%, and 97.79%, respectively, close to the chelating activity of EDTA-2Na (99.84%). In addition, the EC_50_ values of LLP-1, LLP-2, and LLP-3 were 0.71, 0.70, and 0.60 mg/mL, respectively. Studies have shown that all three polysaccharide metabolites exhibit strong *in vitro* free radical scavenging and Fe^2+^ chelating activities. Therefore, it is evident that the efficiency and potency of *L. lancifolium* polysaccharide extracts or active metabolites make them a promising natural antioxidant in pharmaceuticals and functional foods (Xu et al., 2016). Simultaneously, A novel polysaccharide fraction (LP2-1) was isolated and purified from the edible bulbs of 16 *L. lancifoliumL* Thunb by Gao et al. (2015). The study determined the antioxidant activity of LP2-1 through DPPH, Hydroxyl, reducing power, and Fe^2+^ chelating assays. In addition, LP2-1 had DPPH and hydroxyl radicals scavenging activities, and 25 also had the strong reducing power and chelating activity on ferrous ion. These results suggest that LP2-1 has good antioxidant activity and can be used in food industry (Gao et al., 2015).

Additionally, similar *in vitro* experiments were conducted, where Jin et al. (2012) homogenized a fine dried *L. lancifolium* bulb powder sample and extracted it with ultrasonic assistance using an acidified methanol solution (1 M HCl in 80% methanol) at 25 °C for 1 h in an external water bath. Four antioxidant assays, such as DPPH free radical scavenging activity, ABTS radical cation scavenging activity, cupric-reducing antioxidant capacity (CUPRAC) and hydroxyl radical scavenging activity (HRSA) were applied. The results indicated that each bulb extract exhibited strong antioxidant activity, which was positively correlated with the total phenolic content and total flavonol content. This reveals that *L. lancifolium* bulbs can be used as a potential natural antioxidant for food and pharmaceutical applications (Jin et al., 2012). A preparative HSCCC was used to isolate phenylpropanoid glycerides from the bulbs of *L. lancifolium* by Luo et al. (2012). The antioxidant activities of the obtained metabolites were evaluated using three distinct assays: DPPH and ABTS radical scavenging assays, and the ferric-reducing antioxidant power (FRAP) assay. Studies have demonstrated that these metabolites possess extremely strong antioxidant activities from the bulbs of *L. lancifolium* (Luo et al., 2012). In other experiments, Liang et al. (2022) studied the relationship between *Lilium* spp. bulb color and the content of active metabolites, as well as the antioxidant capacity of lilies. Herein, bulbs from 56 wild populations and three cultivars were collected, and their edible characteristics, antioxidant capacities, and pigments were investigated and analyzed. The results showed that phenolic metabolites contributed to the major colors (red, yellow, and white) in *Lilium* spp*.* bulbs. Furthermore, *Lilium* spp. bulbs with darker and redder hues exhibited greater biomass, superior nutritional metabolites, higher active metabolite content, and stronger antioxidant capacity (Liang et al., 2022). Studies have demonstrated that *Lilium* spp. extracts exhibit remarkable antioxidant properties. Their primary active metabolites, including phenolic metabolites and polysaccharides, are responsible for these effects, which are achieved through mechanisms such as free radical scavenging and metal ion chelation. Notably, *Lilium* spp. extracts sourced from different origins display variations in antioxidant capacity, and the extent of their antioxidant effects is closely tied to the concentration of these active metabolites. Given these characteristics, *Lilium* spp. extracts present extensive potential for application in the food and pharmaceutical industries and are anticipated to be developed and utilized as natural antioxidants.

### 5.3 Antidepressant activity

Depression can manifest physically through symptoms such as fatigue, pain, or sleep disturbances (Wang et al., 2024a). Moreover, newer medications tend to be better tolerated than earlier drugs, leading to better patient compliance with treatment (Wang et al., 2024b). As a chronic illness, depression is marked by an increased likelihood of recurrence with each additional episode (Beton-Mysur and Brożek-Płuska, 2025). This often makes long-term maintenance drug therapy necessary. The antiseizure effects of a water extract of Lilii Bulbus (WELB) in mouse model of pentylenetetrazol (PTZ)-induced seizure was evaluated by Park and Cai (2024). The study found that WELB (The animals were treated orally with 500 mg/kg, once daily for 14 days) treatment could prevent PTZ-induced low seizure threshold and high seizure severity. Furthermore, after WELB treatment, the increased or decreased expression of proteins related to ectopic DGCs (Reelin and Dab-1), MFS (Netrin-1, Sema3A, and Sema3F), and their downstream effectors (ERK, AKT, and CREB) in the hippocampus of PTZ-kindled mice were significantly restored. Overall, our findings suggest that WELB is a potential anti-epileptic drug candidate, including convulsive seizures, ectopic DGCs, and MFS (Park and Cai, 2024). Simultaneously, lily aqueous extract is a potential candidate drug for estradiol. In their experiment, Zhou et al. (2019a) evaluated the ameliorative effects of an aqueous extract of lily bulb (AELB) on menopause-associated psychiatric disorders and its underlying mechanisms relative to estrogen therapy. In their experiment, ovariectomized (OVX) mice were administered AELB (1.8 g/kg) or estradiol (0.3 mg/kg) for 5 weeks. The study concluded that AELB exhibits anti-anxiety, anti-depressant, and cognitive-enhancing effects similar to those of estradiol. AELB reversed the OVX-induced decrease in the expression levels of hippocampal nerve growth factor (NGF) and prefrontal glial cell-derived neurotrophic factor (GDNF). Furthermore, AELB also increased the expression levels of ER*β* in the uterus and brain regions. AELB’s efficacy in alleviating menopause-like behaviors is comparable to that of estradiol, suggesting that its metabolites may offer a novel therapeutic avenue for menopause (Zhou et al., 2019a). In another study, Zhou et al. (2019b) sought to further investigate the psychotropic effects of total polysaccharides of lily bulb (TPLB) against anxiety, depression, and cognitive deterioration and the underlying mechanisms in OVX mice using behavioral, neurochemical, molecular, and proteomic approaches in comparison with estrogen therapy. The researchers found that estradiol and TPLB could reduce glutamate levels and NMDAR1 expression in the hippocampus and prefrontal cortex, and increase the p-CaMKII/CaMKII ratio. These findings suggest that TPLB may exert anti-menopausal effects with improved safety profiles, highlighting its significant potential as a promising drug candidate for menopausal syndrome (Zhou et al., 2019b).

Plants of *Lilium* spp. can also play a significant role in the treatment of Alzheimer’s disease, and researchers have begun to explore these applications in recent years. This study by Hui et al. (2024a) aims to investigate the possible mechanism of *L. brownii* extract in treating PD and to compare the efficacy of ethanol and aqueous extracts of *L. brownii*. In this study, mice with PD induced by 1-methyl-4-phenyl-1,2,3,6-tetrahydropyridine hydrochloride were administered *L. brownii* extracts for 30 days, after which the effects of both extracts were evaluated. The study concluded that both extracts of *L. brownii* effectively improved motor dysfunction in MPTP-induced PD mice. Furthermore, they reduced levels of MDA and ferrous ion (Fe^2+^), while increasing levels of SOD and glutathione peroxidase (GSH-Px) in serum. In summary, *L. brownii* shows promise as an effective and safe treatment for PD (Hui, C. et al., 2024b). Depression-like behaviors were successfully modeled in mice following exposure to chronic unpredictable mild stress (CUMS). Ma et al. (2024) further explored the underlying potential mechanisms of a combination therapy through both *in vitro* and *in vivo* experiments. They analyzed the expression levels of COX-2, PGE2, and IL-22, along with microglial activation and neuronal viability/apoptosis in the hippocampus. These analyses revealed that the combination of Longya *Lilium* and Fluoxetine synergistically reduced COX-2 expression, thereby alleviating depression-like behaviors and neuroinflammation in mice. Specifically, the downregulation of COX-2 inhibits the activation of BV-2 microglial cells, mitigates inflammation, and reduces neuronal apoptosis by suppressing the PGE2/IL-22 axis. Thus, the combination of Longya *Lilium* and Fluoxetine inactivates the COX-2/PGE2/IL-22 axis, ultimately relieving neuroinflammatory responses and the associated depression-like behaviors (Ma et al., 2024).

Recently, Yuan et al. (2021) further used Cell Counting Kit-8 assay and Western blotting to evaluate the neuroprotective antidepressant effects of Regaloside A. The results showed the cell survival rate was improved, the phosphorylation levels of brain-derived neurotrophic factor, tyrosine kinase receptor B, phosphatidylinositol three kinase, protein kinase B, and mammalian target of rapamycin were increased after Regaloside A treatment. It could also alleviate the damage of corticosterone in SH-SY5Y cells. This study revealed that phosphatidylinositol-3kinase/protein kinase B/mammalian target of rapamycin signaling mediated by brain-derived neurotrophic factor/tyrosine kinase receptor B may play an important role in the neuroprotective antidepressant effects of Regaloside A (Yuan et al., 2021). Additionally, the neuroprotective potential of an aqueous extract of *L. lancifoliumL* Thunberg (ELL) against corticosterone (CORT)-induced pathophysiology in PC12 cells was reported by Lee et al. (2025). To evaluate the neuroprotective potential of ELL, PC12 cells were pretreated with 50 μg/mL ELL prior to CORT exposure. ELL markedly prevented CORT-induced neuronal death by suppressing the expression of pro-apoptotic proteins, reducing lactate dehydrogenase release, and attenuating reactive oxygen species production while preserving intracellular adenosine triphosphate levels. Moreover, ELL alleviated CORT-triggered endoplasmic reticulum (ER) stress, as evidenced by diminished unfolded protein responses, stabilized intracellular Ca^2+^, inhibition of mitochondrial permeability transition pore opening, and maintenance of mitochondrial membrane potential. Collectively, these findings indicate that ELL confers neuroprotection by blocking apoptosis via suppression of ER stress and preservation of mitochondrial integrity (Lee et al., 2025).

**FIGURE 6:**
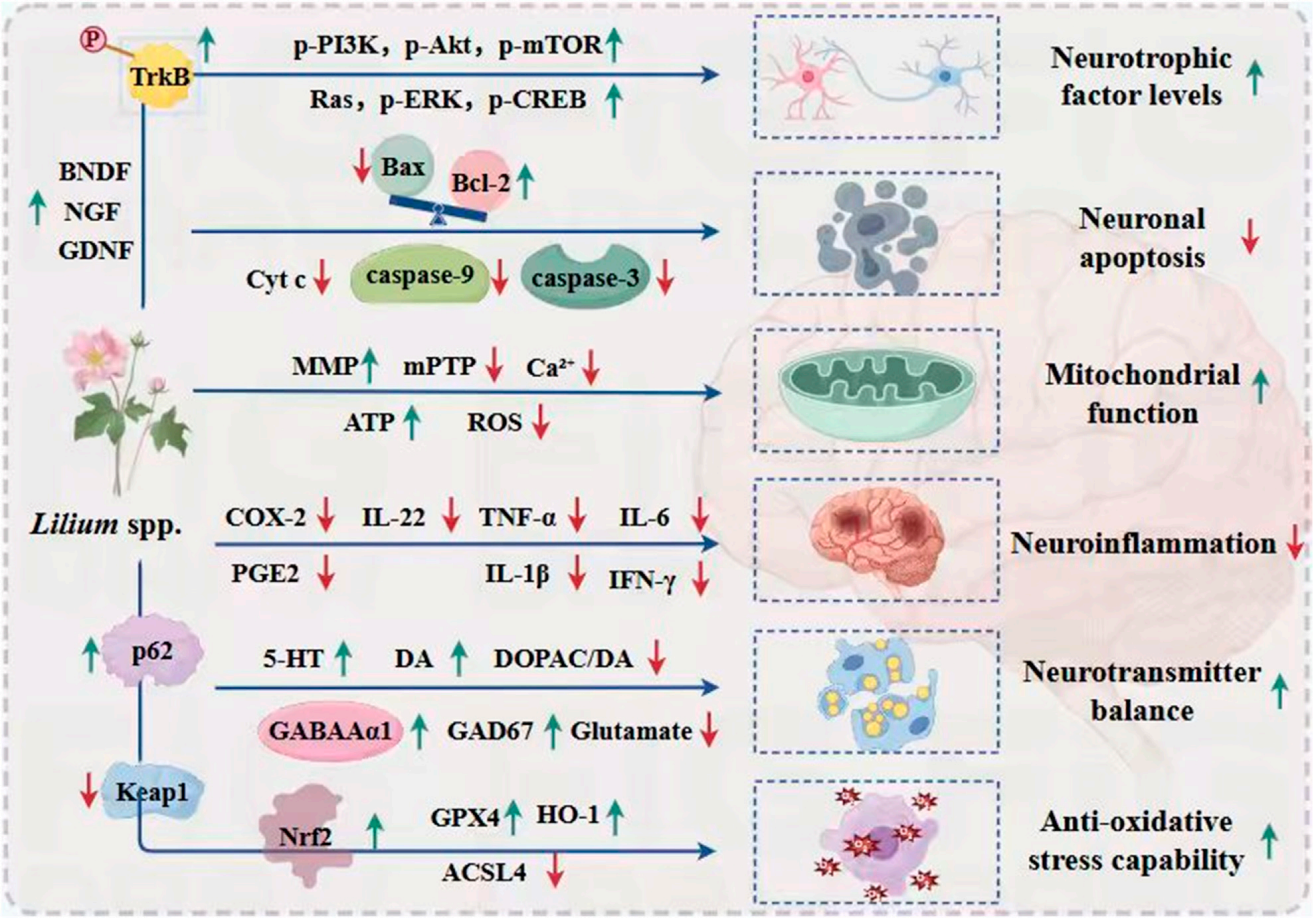
Schematic representation of underlying molecular mechanism displaying the antidepressant activity of the *Lilium spp.*

To summarize, *Lilium* species extracts appear highly promising as neuroprotective agents and as therapeutics for neuropsychiatric conditions. They regulate the BDNF/TrkB/PI3K/Akt/mTOR signaling pathway to enhance neurotrophy and anti-apoptosis effects; inhibit the COX-2/PGE2/IL-22 inflammation axis to alleviate neuritis and microglial activation; regulate the NMDAR1/CaMKII pathway to balance glutamic acidergic neurotransmission; upregulate the expression of neurotrophic factors such as NGF and GDNF through ER*β*-mediated Estrogens-like effects; and exert anti-Depression and neuroprotective effects by inhibiting endoplasmic reticulum Stress, maintaining mitochondrial membrane potential and ATP levels, and blocking the apoptosis pathway. These different mechanisms make *Lilium* extracts promising for the development of new therapeutic drugs or adjuvant therapies. The regulatory mechanism of the *Lilium* genus governing the mRNA and protein expression levels of antidepressant associated factors are illustrated in Figure 6.

### 5.4 Antitumor activity

Chemotherapy is a traditional approach that has long played a vital role in tumor treatment. However, current clinical anti-tumor drugs are often plagued by drawbacks such as limited efficacy and significant side effects (Lei et al., 2025). Moreover, the development of new anti-tumor drugs is a lengthy process that demands substantial resources. In contrast, the active metabolites in traditional Chinese medicine (TCM) are increasingly recognized for their potential anti-tumor effects, which are often characterized by fewer side effects (Ren et al., 2026). Zhou J. et al. (2024) firstly isolated four unprecedented steroidal saponins from lily bulbs, together with five known congeners. Evaluation of their cytotoxic potential against MCF-7, MDA-MB-231, HepG2, and A549 cell lines revealed that metabolite 9 selectively inhibited MCF-7 cells with an IC_50_ of 15.2 μM. Moreover, metabolite 6 was found to trigger G_2_/M-phase arrest and apoptosis in HepG2 cells (Zhou J. et al., 2024). In another study, Zhang et al. (2020) investigate the effects of total saponins from *L. lancifoliumL* (TSLL) on proliferation, apoptosis and migration of human gastric carcinoma cells lines SGC-7901 and HGC-27 and its underlying mechanism. The study concludes that TSLL inhibits the proliferation of stomach cancer cells by inhibiting the level of proliferating cell nuclear antigen (PCNA) and increasing the level of p21. TSLL induces apoptosis by up-regulating the expression of pro-apoptotic protein Bax and down-regulating the expression of anti-apoptotic protein Bcl-2. Meanwhile, TSLL significantly inhibits cell migration and invasion, reduces the expression of matrix metalloproteinase-2 (MMP-2), and increases the expression of tissue inhibitor of metalloproteinase-1 (TIMP-1). In summary, TSLL can be used as an alternative or adjuvant to chemotherapeutic drugs for cancer treatment, revealing that TSLL may be a promising candidate for preventing and inhibiting the growth of stomach cancer cells (Zhang et al., 2020).

In addition, a water-soluble polysaccharide LP60-1 was isolated and purified from *L. davidii* var. unicolor Cotton by Zhang M. et al. (2022). Furthermore, the anti-tumor activity was assessed by MTT method. The study concluded that LP60-1 has significant anti-tumor activity against MDA-MB-231, A549, HepG2, and MCF7 cells. In summary, LP60-1 can be studied and developed as a potential novel anti-cancer drug (Zhang M. et al., 2022). Concurrently, Partovi et al. (2023) aimed to evaluate the anti-proliferative activity of *Lonicera nummularifolia*, *Lilium ledebourii*, *Campsis radicans*, and *Parthenocissus quinquefolia* extracts. These extracts were obtained from fresh leaves and bulbs of the plants through maceration in the dark. Following solvent separation, the extracts were introduced into a culture medium containing G292, A431, and KB cancer cells, along with HGF-1 normal cells, for cytotoxicity, cell cycle, and apoptosis assays. The study concluded that the methanol extract activated p53 protein-induced apoptosis by upregulating the expression of BID/MAPK14 and downregulating the expression of MDM2/BCL2/MYC (Partovi et al., 2023). Research indicates that steroidal saponins, structural polysaccharides, and other active metabolites of *Lilium* spp. arrest the cell cycle at the G_2_/M phase and upregulate p21. They also block PCNA-mediated proliferation signals and MMP-2-dependent migration and invasion. Furthermore, these metabolites suppress anti-apoptotic proteins such as Bcl-2 and MDM2, activate the p53-Bax-caspase pathway to induce apoptosis, synergistically increase ROS and MAPK14 stress signals, and enhance immune recognition. These actions demonstrate their potential as candidates for novel anticancer therapies.

### 5.5 Hypoglycemic activity

Diabetes mellitus is a group of metabolic disorders defined by hyperglycemia resulting from defects in insulin secretion, insulin action, or both. The chronic hyperglycemia in diabetes is associated with long-term damage, dysfunction, and failure of various organs, particularly the eyes, kidneys, nerves, heart, and blood vessels (Haider et al., 2025). The Caco-2 cell was used to study the glucose absorption regulation and mechanism of kaempferol, caffeic acid and quercetin-3-O-*β*-D-galactoside in *L. lancifoliumL* Thunb *in vitro*. Glucose oxidase-peroxidase (GOD-POD) method was used to measure glucose consumption in supernatant. Western blotting and quantitative real-time PCR were used to detect the protein expression and mRNA transcription. The results showed that caffeic acid and quercetin-3-O-*β*-D-galactoside could significantly promote the absorption of glucose by normal Caco-2 cells. It can also significantly promote the uptake of glucose tracer 2-NBDG by Caco-2 cells, as well as the protein expression levels and mRNA transcription of SGLT1 and GLUT2. The study reveals that its mechanism of action may be related to promoting the protein expression levels and mRNA transcription of SGLT1 and GLUT2 (Xu et al., 2021). In other *in vitro* studies, Zhu et al. (2014) used a bioassay-guided approach to explore the potential anti-hyperglycaemic *L. brownii* fraction (s). Their anti-hyperglycaemic activities were further confirmed in human hepatoma HepG2 cells and murine 3T3-L1 preadipocytes. The study concluded that *L. brownii* fraction (SGL) could increase glucose consumption in HepG2 cells and 3T3L1 adipocytes by 1.61- and 2.13-fold, respectively, and enhance 3T3-L1 preadipocyte differentiation (Zhu et al., 2014).

In another study, Hui et al. (2024c) sought to elucidate the fine structure, morphology, and thermal properties of a galactoglucan (BHP-2) derived from Lanzhou lily bulbs. The *in vitro* hypoglycemic potential of BHP-2 was evaluated by assessing its inhibitory effects on *α*-glucosidase and *α*-amylase. The results indicated that BHP-2 exerted competitive inhibition against *α*-amylase and mixed non-competitive inhibition against *α*-glucosidase, with respective IC_50_ values of 0.31 mg/mL and 0.18 mg/mL. These values were comparable to those of acarbose (0.27 mg/mL for *α*-amylase and 0.12 mg/mL for *α*-glucosidase). Overall, BHP-2 demonstrated favorable thermal stability and a significant hypoglycemic effect (Hui, H. et al., 2024c). Similar investigations have previously been carried out. In one such study, Hui et al. (2024d) assessed the hypoglycemic effects of O-acetyl mannoglucan (BHP-1) obtained from Lanzhou lily bulbs. The findings indicated that BHP-1 demonstrated substantial inhibitory activity against *α*-glucosidase and moderate inhibitory activity against *α*-amylase within the tested concentration range. These results underscore the significant potential of BHP-1 in reducing blood sugar levels (Hui, H. et al., 2024d).

Additionally, the protective effect of *L. lancifoliumL* polysaccharides (LLP) on streptozotocin (STZ, Dose of 200 mg/kg, gavage, for 28 days)-induced diabetic mice and possible mechanism were investigated by Zhang et al. (2014). The activities of antioxidant enzymes superoxide dismutase (SOD), glutathione peroxidase (GPx), and catalase (CAT) in serum, liver, and kidney of STZ-induced diabetic mice were measured, and the level of malondialdehyde (MDA) was reduced. It was concluded that oral administration of LLP could significantly reduce blood glucose levels in STZ-induced diabetic mice, increase body weight, significantly increase the activities of SOD, GPx, and CAT in serum, liver, and kidney of STZ-induced diabetic mice, and reduce MDA levels. At the same time, after LLP administration, pancreatic damage was significantly repaired, and the integrity of islet cells and tissues was improved. In conclusion, this study demonstrates that LLP exerts hypoglycemic and protective effects on STZ-induced diabetic mice by reducing oxidative stress and maintaining pancreatic islet integrity (Zhang et al., 2014). In conclusion, *Lilium* spp. extracts hold considerable promise for modulating blood glucose via a multi-targeted mode of action. They upregulate SGLT1/GLUT2 expression in Caco-2 cells, promoting intestinal glucose absorption; competitively inhibit *α*-amylase and non-competitively inhibit *α*-glucosidase, delaying carbohydrate breakdown; increase SOD, GPx, and CAT activity and decrease MDA, mitigating pancreatic islet oxidative damage and maintaining *β*-cell integrity; and enhance 3T3-L1 preadipocyte differentiation, improving insulin sensitivity. These mechanisms indicate that *Lilium* spp. extracts could be promising candidates for developing hypoglycemic drugs or functional foods.

### 5.6 Immune enhancement activity

Innate immunity forms the host’s first - line of defense against infectious agents, providing a rapid response to pathogen invasion. Key cellular metabolites of this arm of the immune system include neutrophils and macrophages. These cells are equipped to perform phagocytosis, engulfing and eliminating invading pathogens. Simultaneously, they release inflammatory mediators and cytokines to signal the presence of infection and orchestrate the immune response. Macrophages, in particular, serve as critical mediators in both innate and adaptive immunity. They are capable of secreting a diverse array of molecules such as inflammatory mediators, cytokines, chemokines, as well as stress response and anti - apoptotic proteins, thus playing a multifaceted role in immune regulation (Brooks et al., 2026). In a recent study, Pan et al. (2017) explored the *in vitro* immune-enhancing activity of the water-soluble polysaccharide fraction (LLP-1A) in macrophages and the underlying molecular mechanism. The study found that in terms of the effect on macrophages, LLP-1A enhanced the phagocytic activity of macrophages, induced the production of NO, and was dose-dependent. In addition, it also induced the expression of cytokines interleukin-6, monocyte chemoattractant protein 1, tumor necrosis factor-*α*, and interleukin-1*β*. In terms of molecular mechanism, LLP-1A increased the protein expression of toll-like receptor 4 in RAW 264.7 cells, as well as the phosphorylation of nuclear factor κB kinase inhibitor, NF-κB inhibitor, and nuclear factor κB. This study suggests that LLP-1A may have immunomodulatory functions and warrants further investigation to clarify its potential utility in immunization-related diseases (Pan et al., 2017). Simultaneously, in study, crude polysaccharides were extracted from bulblet of *L. brownii* by aqueous alcoholic precipita-tion, and eluted on a DEAE-52 column to obtain *L. brownii* polysaccharide (LBP) by Li X. J. et al. (2024). *In vitro* experimental results showed that LBP has good biocompatibility, and a low dose of 5 μg/mL LBP can significantly upregulate the expression of TNF-α, iNOS, IL-6, IL-1β, and toll-like receptor family (TLRs) mRNA in RAW 264.7 cells. In summary, LBP plays an important role in immunomodulation, and it may exert its immunisation activity through TLRs family receptors (Li X. J. et al., 2024).

In addition, Li et al. (2021) delved into the immunoregulatory potential of selenized polysaccharides from lily bulb (sLP). *In vitro* experiments revealed that sLP significantly enhanced the phagocytic activity of RAW 264.7 cells, elevated the levels of interleukin (IL)-1*β* and IL-2, boosted acid phosphatase activity, and upregulated the surface expression of CD86 and CD80 molecules. *In vivo* studies further demonstrated that sLP positively influenced the immune system by improving the indices of immune organs, increasing the serum levels of interferon-*γ*, IL-6, immunoglobulin (Ig)G, and IgM, and stimulating lymphocyte proliferation (Li et al., 2021). In summary, *Lilium* spp. species extracts display remarkable immunomodulatory activity. They engage and activate macrophage TLR4/TLR family receptors, inducing IKK-NF-κB axis phosphorylation, upregulating CD80/CD86 co-stimulatory molecules, and significantly boosting phagocytic capacity and the expression of key inflammatory mediators, including NO, TNF-α, IL-1β, IL-6, and iNOS. Furthermore, they promote dendritic cell maturation, increase serum levels of IFN-γ, IL-2, IgG, and IgM, and enhance antigen presentation. Ultimately, they increase the immune organ index and lymphocyte proliferation rate, synergistically enhance acid phosphatase activity, and establish a positive immunomodulatory feedback loop. Overall, *Lilium* spp*.* species extracts are highly promising for developing new immunomodulatory agents.

### 5.7 Maintain lung function activity

Pneumonia, a prevalent lower respiratory tract infection, primarily affects the alveoli and distal bronchioles, and can progress to severe complications such as acute respiratory distress syndrome (ARDS) and sepsis (Garvey, 2024; Han and Mallampalli, 2015). Current treatment strategies mainly revolve around anti - infective therapy and prevention of complications, with common medications including antitussives, antipyretic analgesics, and antibiotics. However, the growing concern of drug resistance caused by the overuse of these medications (Li et al., 2023), coupled with their potential adverse effects, has highlighted the urgent need for novel therapeutic approaches. In this context, the extraction of natural bioactive metabolites from plants, which are safe, effective, and free from undesirable side effects, offers a promising alternative for the treatment and rehabilitation of pneumonia patients. In this study, the effect of *L. lancifolium* Extract on Pulmonary Inflammatory Response in CS-Exposed Mouse Model by Lee et al. (2013). Water extract of *L. lancifolium* root was fed to C57BL/6 mice prior CS exposure every day for 3 weeks. The study used real-time fluorescence quantitative PCR, ELISA, or Western blot to detect relevant inflammatory factors, TNF-α, IL-6, IL-1β, monocyte chemoattractant protein-1 (MCP-1), and matrix metalloproteinase-12 (MMP-12). In the CS-exposed mouse model, we found that *L. lancifolium* extract could reduce inflammatory cells (macrophages and neutrophils), pro-inflammatory cytokines (TNF-α, IL-6, IL-1β), chemokines (MCP-1), and proteases (MMP-12). It also reduced airway dilation in CS-exposed mice. The above studies conclude that *L. lancifolium* extract may be a candidate drug for the treatment of CS-induced lung inflammation and emphysema (Lee et al., 2013). Meanwhile, Eom et al. (2023) investigated the protective effect of *Lilium* longiflorum Thunb (LLT) bulb extract fermented with *Lactobacillus* acidophilus 803 in COPD mouse models induced by cigarette smoke extract (CSE) and porcine pancreas elastase (PPE). The study showed that oral administration of the fermentation product (LS803) (at a Dose of 100 and 200 mg/kg, gavage, for 22 days) could inhibit the production of inflammatory mediators and the infiltration of immune cells (including neutrophils and macrophages), thereby exerting a protective effect on lung injury. Furthermore, LS803 significantly inhibited the increased production of IL-6 and MIP-2 after CSE and LPS stimulation by inhibiting the activity of NF-κB in mouse peritoneal macrophages. In summary, the fermentation product LS803 demonstrated preventive and alleviating effects on pulmonary inflammation in this model, providing preliminary evidence that warrants further studies to clarify its potential for managing pulmonary inflammatory diseases (Eom et al., 2023).

In their study, Liu R. et al. (2025) isolated a novel polysaccharide (L005-B) from *L. lancifolium* and characterized its Anti-pulmonary fibrosis properties. *In vitro* experiments demonstrated that L005-B effectively counteracted TGF-*β*1-induced epithelial-mesenchymal transition (EMT) in A549 alveolar epithelial cells, as evidenced by reversal of E-cadherin downregulation, and suppression of *α*-smooth muscle actin (*α*-SMA) and vimentin overexpression. Using a bleomycin-induced pulmonary fibrosis mouse model, the team further observed that L005-B (doses ranging from 0 to 1,000 μg/mL for 48 h) administration significantly reduced fibrotic markers (*α*-SMA, COL1A1, and fibronectin) while restoring E-cadherin expression *in vivo*. These findings collectively position L005-B as a promising therapeutic candidate for pulmonary fibrosis (Liu R. et al., 2025). In addition, the biological effects and mechanisms of Lily extract on LPS-induced pneumonia mice were explored by Sun et al. (2024). In the experiment, LPS was used to construct a mouse pneumonia model, and *L. brownii* var. viridulum extract was extracted via ultrasonic alcohol extraction. The study concluded that treatment with low-dose and high-dose Lily extract (100 mg/kg, 200 mg/kg) for 48 h significantly reduced lung tissue inflammatory cell infiltration, pro-inflammatory factor (IL-6, IL-1β, TNF-α) mRNA expression levels, and inflammation-related protein (IL-6, NF-κB P65) protein levels. Furthermore, Lily extract significantly improved the assembly and motor function of cilia, and the expression levels of cilia-related genes (Ttc21a, Cfap45, etc.) and proteins were significantly upregulated (Sun et al., 2024).

In other experiments, Khan et al. (2024) explored enhancing the active metabolites in *L. davidii* (Lanzhou Lily) bulbs by fermenting them with Limosilactobacillus fermentum GR-3, a strain isolated from Jiangshui. The fermented lily bulb fraction (LFB + GR-3) was used to treat mice exposed to carbon black nanoparticles (CBNPs). Results showed that CBNP deposition and lung tissue damage were markedly reduced in the LFB + GR-3 group. Meanwhile, TNF-α, IL-10, and IL-6 levels increased by 6.9, 4.3, and 7 times, respectively, in the CBNP-exposed group. Additionally, probiotic-fermented lily bulbs may aid in treating lung infections (Khan et al., 2024).

**FIGURE 7:**
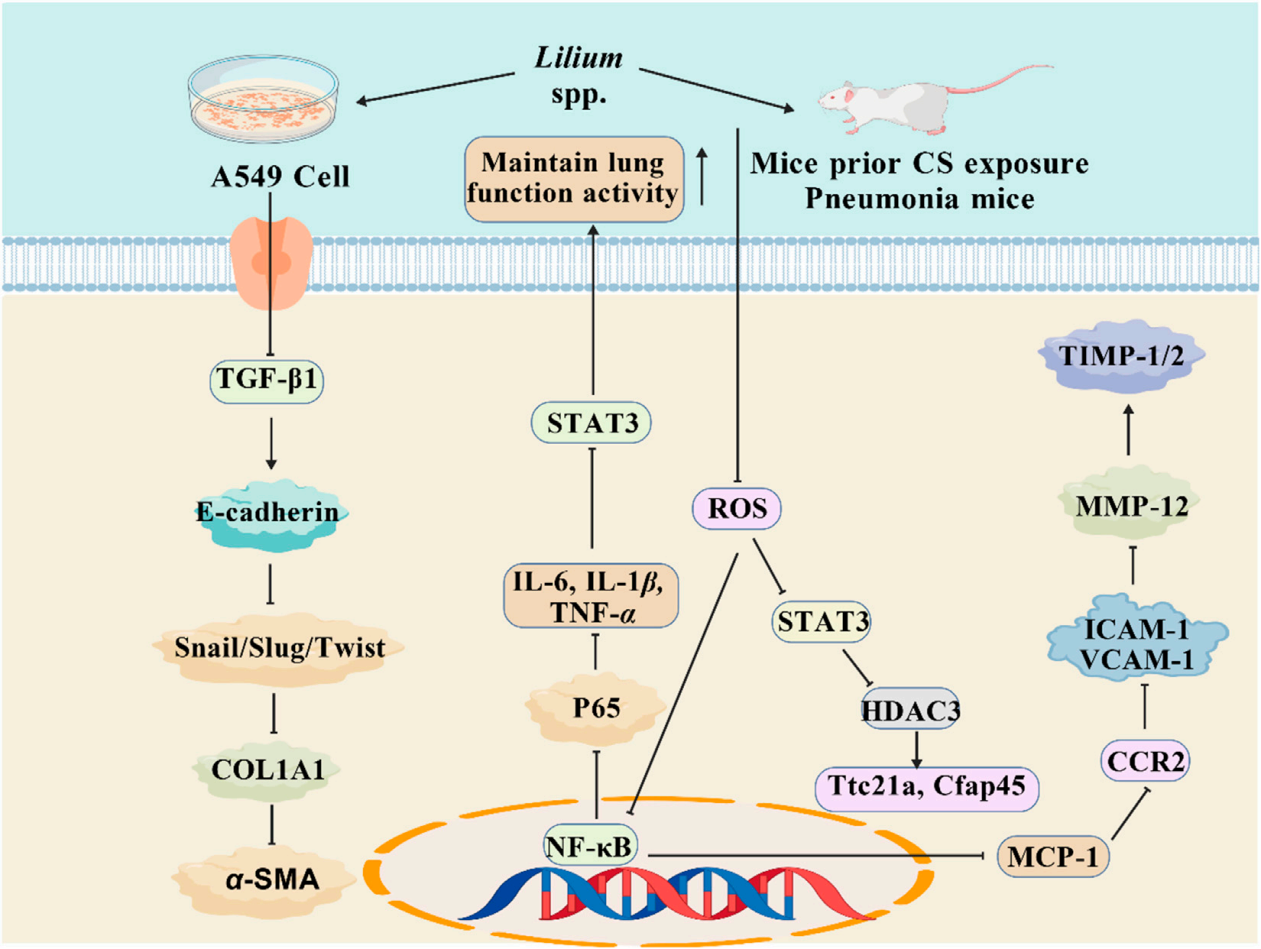
Schematic representation of underlying molecular mechanism displaying the maintain lung function activity of the *Lilium spp*.

Research indicates that extracts from *Lilium* spp. species hold significant potential for mitigating pulmonary inflammation and fibrosis. These block the NF-κB signaling cascade, significantly downregulate pro-inflammatory mediators such as TNF-α, IL-6, IL-1β, and MMP-12, and reduce neutrophil and macrophage count infiltration; inhibit TGF-*β*1-driven epithelium-mesenchymal transition (EMT), reverse E-cadherin downregulation, and reduce *α*-SMA, COL1A1, and Fibronectin expression, thereby alleviating the pulmonary fibrosis process; meanwhile, upregulate cilia-related genes such as Ttc21a and Cfap45, and repair airway epithelium cilia structure and motor function. Thus, *Lilium* spp. extracts show promise as therapeutic candidates for pulmonary inflammatory diseases, including COPD, pneumonia, and pulmonary fibrosis, and merit further exploration of their clinical applications. The regulatory mechanism of the *Lilium* genus governing the mRNA and protein expression levels of maintain lung function associated factors are illustrated in Figure 7.

### 5.8 Gastrointestinal protection activity

Non-steroidal anti-inflammatory drugs (NSAIDs) are among the most widely used medications currently available. Their use is associated with gastrointestinal toxicity, affecting both the upper gastrointestinal tract (peptic ulcer disease) and the lower gastrointestinal tract (NSAID-induced enteropathy). Therefore, clinicians should avoid prescribing NSAIDs for long-term therapy or at high doses (Hijos-Mallada et al., 2022). In this study, Li and co-workers (2025) isolated a novel RG-I like pectin, L01-B1, with an average molecular weight of 43.9 kDa from the flowers of *L. lancifolium*. They elucidated its macromolecular structure and function in the human gut microbiota, revealing that the backbone of L01-B1 consists of 1,6-*β*-galactan and RG-I pectin. *In vitro* evaluation of its effects on human gut microbiota demonstrated that L01-B1 altered microbial composition and increased the abundance of *Bifidobacterium longum*. Additionally, L01-B1 may be degraded by two types of enzymes, potentially altering gut microbiota metabolism (Li M. et al., 2025). Moreover, The results of this study suggest that L01-B1 may be beneficial to human gut health by modulating Bifidobacterium longum.

The effect of lily bulbs on the microecological characteristics of intestinal microbiota and enzyme activities in normal micewere investigated by Wu and co-workers (2021). In the experiment, the administration group mice were given 0.15 g/mL lily bulb solution, intragastrically administered twice daily, and the control group mice were given the same volume of sterile water. After 49 days, intestinal contents and mucosa of each group of mice were collected to analyze the characteristics of intestinal flora and enzyme activity. The experiment proved that lily bulb can significantly increase the activity of amylase in intestinal contents and improve the digestion and absorption of food. In addition, lily bulb stimulates intestinal epithelial cells to secrete lactase, increases mucosal lactase activity, and maintains intestinal mucosal immunity. At the same time, it can promote the growth of *Lactobacillus* and Bifidobacterium in the intestine of normal mice, inhibit the growth of total bacteria in the intestine, and maintain the intestinal mucosal barrier. In summary, this study may help reveal the potential of lily bulb as a functional food and further strengthen the research on its active metabolites for improving intestinal microecology (Wu et al., 2021). In summary, an aqueous solution of lily bulb significantly enhances the activity of amylase and lactase, improving nutrient digestion and absorption while maintaining mucosal immunity. Furthermore, it promotes the colonization of lactobacilli and bifidobacteria, inhibits the overgrowth of opportunistic pathogens, and strengthens the intestinal mucosal barrier function. This multi-target regulatory mechanism provides a theoretical basis for developing lily-derived functional foods or microbial agents as alternatives to NSAIDs and their associated damage.

### 5.9 Relieve joint pain activity

Rheumatoid arthritis (RA) is a systemic chronic inflammatory disease characterized by widespread synovitis, which leads to the erosion of articular cartilage and marginal bone, and ultimately joint destruction (Hussein et al., 2025; Jeon et al., 2024). NSAIDs are currently commonly used to treat Osteoarthritis (OA) (Dixit et al., 2025). However, some studies have found that long-term use of non-steroidal anti-inflammatory drugs can cause various health problems, including nephrotoxicity (Baker and Perazella, 2020) and gastrointestinal diseases (Domper Arnal et al., 2022). Arthritis is a common geriatric disease characterized by joint pain, limited mobility, and significantly reduced quality of life. HY-LL was prepared by (Jeon et al., 2024) using 50% ethanol extraction from the Dried *L. lancifoliumL* bulbs. Several subjects were recruited for this trial and randomly assigned to the HY-LL group or the placebo control group, with oral administration of tablets twice daily for 12 weeks. The effects of HY-LL on the degree of joint pain and quality of life were systematically evaluated during the study. The results showed that continuous administration of HY-LL for 12 weeks significantly improved the overall quality of life of the subjects. This confirms that 12 weeks of HY-LL intervention can relieve joint pain and improve quality of life, suggesting the potential value of *L. lancifoliumL* bulbs ethanol extract in the treatment of arthritis pain (Jeon et al., 2024).

In this study, OA was induced by experimenters in 2-year-old dogs through resection of cranial cruciate ligament and lateral collateral ligament. Inflammatory cytokines, enzymes, lameness score, radiology, and histological changes were assessed. The experiment concluded that long-term oral therapy with *L. lancifolium* (dosage of 60 mg/kg, administered orally once daily for 12 weeks) alleviated inflammation and increased histological damage. *L. lancifolium* treatment effectively reduced cytokines, such as interleukin-6, metalloproteinase-9, leukotriene-4, prostaglandin, and cyclo-oxygenase, suggesting the potential to minimize inflammatory reactions in OA (Cho et al., 2022).

In conclusion, *Lilium* extract alleviates joint pain and dysfunction by downregulating inflammatory mediators and improving joint function. It suppresses the expression of key inflammatory factors, including IL-6, MMP-9, LTB_4_, PGE_2_, and COX-2, thereby reducing synovitis and cartilage degradation, with no significant adverse effects observed. This study provides robust clinical and animal evidence supporting the development of *Lilium* plants into long-acting, safe interventions for arthritis, potentially replacing or complementing traditional NSAID therapies.

### 5.10 Other effects

#### 5.10.1 Myoprotective

Myalgia is a common symptom of various neuromuscular diseases. It is observed in metabolic muscle diseases, inflammatory muscle diseases, muscular dystrophies, and myotonic disorders. Myalgia can lead to a significant decline in the patient’s quality of life (Vasvit et al., 2025). Kim et al. (2024) evaluated the effectiveness of *L. brownii* ethanol extract (LBE) in a dexamethasone (DEX)-induced muscle atrophy model and elucidated its mechanism of action through muscle transcriptome analysis. The effects of citric acid on the viability of C2C12 myoblasts and the density of myotubes, as well as the effects of DEX treatment with or without on C2C12 myotube differentiation, were also investigated. The study concluded that LBE (The animals were administered doses of 100 and 300 mg/kg via gavage, once daily for 10 days) pretreatment had a protective effect on rat C2C12 myoblasts and increased the muscle density of rat C2C12 myotubes. LBE exhibited strong free radical scavenging and antioxidant activity in C2C12 myoblasts. In summary, LBE inhibited muscle protein degradation, preserved the muscle tissue microenvironment, reduced skeletal muscle loss, and maintained muscle function caused by DEX chronic toxicity by protecting muscle cells from various stress conditions and the effects of DEX itself. Research indicates that LBE shows preliminary promise for mitigating glucocorticoid-induced muscle atrophy, although confirmatory studies are still needed (Kim et al., 2024).

#### 5.10.2 Anti-melanogenesis activity

Melanin is synthesized within oval-shaped organelles called melanosomes. These melanosomes are produced by dendritic melanocytes in the epidermal basal layer, representing only 1% of the total cellular composition (Ning et al., 2025). Several *in vivo* and *in vitro* studies have demonstrated the significant anti-Melanogenic Effects of *L. lancifoliumL* Root. Park et al. (2023) studied the anti-melanogenic effects of *L. lancifoliumL* extract (LRE) on B16F10 cells, and concluded that LRE effectively reduced melanin production in a dose-dependent manner without causing cytotoxicity. Meanwhile, LRE decreased cellular tyrosinase activity and the mRNA and protein levels of tyrosinase, Tyrp1, and Tyrp2 in a dose-dependent manner, revealing the potential of LRE to effectively exert anti-melanin effects by inhibiting the expression of core melanogenic enzymes in B16F10 cells (Park et al., 2023).

#### 5.10.3 Anti-insomnia activity

Insomnia is defined as difficulty in sleeping. As a highly prevalent and chronic disabling disease, it places a significant health and economic burden on both individuals and society. Consequently, medicinal plants are receiving increased attention as potential sedative agents, due to their diverse array of natural bioactive metabolites and comparatively fewer side effects (Yan et al., 2025). Twenty-five water-soluble metabolites were isolated from the bulbs of *L. davidii* var. unicolor, including two metabolites termed *Lilium*tides A and B (155–156) by Zhang H. et al. (2022). Compared with the blank control group, the *Lilium*tide A decreased sleep latency and significantly increased the sleep time. In conclusion, *Lilium*tide A could be investigated as a natural anti-insomnia lead metabolite in the pharmaceutical and food industries (Zhang H. et al., 2022). Simultaneously, Si et al. (2022) delved into the molecular mechanism underlying the insomnia-alleviating effects of *L. brownii* (LB) bulb. An insomnia model was established by administering p-chlorophenylalanine (PCPA) to rats via intraperitoneal injection, alongside oral treatment for 7 days at a dose of 598.64 mg/kg. The research team assessed the levels of 5-hydroxytryptamine (5-HT), norepinephrine (NE), and melatonin (MT), examined the expression of GABAA, 5-HT1A, and MT receptors, and analyzed pathological changes within the hypothalamus. The findings revealed that LB treatment significantly counteracted the adverse effects induced by PCPA in the rats. Notably, it enhanced the abundance and diversity of intestinal flora and modulated the fecal metabolic phenotype. Specifically, LB administration led to increased levels of 5-HT (8.14 ng/mL) and MT (16.16 pg/mL), upregulated 5-HT1A and GABAA receptors, reduced NE levels (0.47 ng/mL), and improved the pathological conditions of hypothalamic cells. Overall, the study highlights the promising potential of LB as a health food for insomnia treatment (Si et al., 2022).

#### 5.10.4 Alleviate obesity activity

Obesity, particularly when characterized by excessive visceral fat distribution, is associated with multiple alterations in hormone, inflammation, and endothelial levels. These changes stimulate several other mechanisms, ultimately leading to hypertension and, consequently, increased cardiovascular morbidity (Kar et al., 2025). Park et al. (2023) explore the effects of lily bulbs’ polyphenols (LBPs) on oxidative stress and lipid metabolism. *In vitro* studies revealed that LBPs mitigated the disruption of mitochondrial membrane potential, inhibited the production of reactive oxygen species, reduced oxidative stress, and decreased lipid accumulation in oleic acid-induced HepG2 cells. *In vivo* studies showed that LBPs (doses of 150 and 300 mg/kg via gavage for 11 weeks) significantly inhibited weight gain in mice, reduced serum and liver lipid levels, and improved oxidative damage in a dose-dependent manner. Furthermore, LBPs also ameliorated hepatic steatosis, inhibited the expression of hepatic lipogenesis-related genes (SREBP-1c, FAS, ACC1, and SCD-1), and promoted the expression of lipolysis genes (SRB1 and HL) and lipid oxidation genes (PPAR*α* and CPT-1), highlighting the significant potential of LBPs in the prevention and treatment of obesity and obesity-related diseases (Kan et al., 2021).

#### 5.10.5 Hepato protection activity

Long-term ethanol consumption can lead to liver damage and unfavorable blood lipid profiles in humans. Toxic acetaldehyde, which is formed from alcohol under the catalysis of alcohol dehydrogenase, causes various adverse reactions such as thirst, vomiting, fatigue, headache, and abdominal pain (Zhao et al., 2021). Non-alcoholic fatty liver disease (NAFLD) is intricately connected to the gut microbiota. Recent advancements highlight that leveraging natural polysaccharides as prebiotics is emerging as a highly promising therapeutic avenue for alleviating NAFLD (Younossi, 2019). In this study, Li Z. et al. (2025) explored the therapeutic potential of Lanzhou Lily polysaccharides (LLP) for high-fat diet-induced non-alcoholic fatty liver disease (NAFLD). The findings showed that LLP administration significantly attenuated NAFLD, as evidenced by substantial reductions in lipid accumulation and liver function markers in HFD-induced NAFLD mice. Specifically, LLP treatment led to decreased serum levels of TG, TC, HDL-C, and LDL-C. Moreover, histological analysis using hematoxylin and eosin (H&E) and Oil Red O staining demonstrated that LLP effectively alleviated hepatic steatosis. Additionally, LLP suppressed the expression of pro-inflammatory cytokines TNF-α and IL-1β, reducing hepatic inflammation. Overall, LLP exhibited potential therapeutic effects on NAFLD and may hold promise for its clinical application (Li Z. et al., 2025).

## 6 Toxicological studies

The genus *Lilium* spp. has a medicinal and edible history of over two thousand years, and modern research has also confirmed its multi-target pharmacological activity, but the label of food-drug homology is not equivalent to absolute safety. With the expansion of clinical doses of *Lilium* preparations and the increase in the application population, systematically sorting out its toxicological profile, clarifying species differences and long-term exposure risks has become an unavoidable part of ensuring rational drug use and functional food development. Toxicological studies of *Lilium* plants began as early as the Eastern Han Dynasty. The “Shen Nong’s Herbal Classic” has listed *L. brownii var*. viridulum and *L. brownii* F.E.Br. ex Miellezis as “none-toxic” top grade, and later generations of Materia Medica followed this statement. The current “Chinese Pharmacopoeia” stipulates that the clinical dose of dried *Lilium* is 6–12 g/d; comprehensive ancient and modern literature shows that the oral safety window can reach 5.55–60 g, and external use does not exceed 65 g, and no acute toxicity record has been found within this range (Wang et al., 2020). In modern research, there are few toxicological studies on *Lilium*, and no cases of *Lilium* poisoning have been found so far, but the possibility of *Lilium* causing chronic poisoning cannot be ruled out (Song et al., 2023). At the same time, *Lilium* plants have species differences in animal toxicity, and rodents and rabbits are highly tolerant to *Lilium*: no toxicity renal or death occurred in mice and rats with gavage equivalent dose ≥1.5 times the weight; dogs and cats showed obvious sensitivity. Fitzgerald et al. reported that dogs developed vomiting and gastrointestinal hemorrhage after accidental ingestion (Fitzgerald, 2010); Langston et al. recorded a case of a cat that died of acute kidney failure within 48 h after eating L. *tigrinum* (Langston, 2002).

In summary, *Lilium* has no significant acute toxicity to humans and has good safety within the dose and course of treatment specified in the Chinese Pharmacopoeia; however, it has toxicity renal to cats and dogs, and clinical attention should be paid to pet contact. Long-term excessive use may pose potential colchicine-like risks, and systematic toxicology data are urgently needed to support its wider clinical application.

## 7 Potential applications

As mentioned above, *Lilium* spp., an important plant resource, is mainly distributed throughout the Northern Hemisphere, including Asia, North America, and Europe (Zhou N. et al., 2024). The east coast of Asia, the west coast of North America, and the Mediterranean region are particularly rich in *Lilium* spp. resources. Furthermore, this plant commands a large market and possesses significant economic and medicinal value (Munafo and Gianfagna, 2015). *Lilium* spp. is rich in bioactive metabolites and possesses significant bioactivities. It holds considerable potential for applications in functional food, health products, and the pharmaceutical industry. Hence, developing nutritious and more stable *Lilium*-related products is of great significance.

In the realm of food technology, a study explored how natural flower extracts from Prunus persica, Rosa chinensis, and *Lilium* davidii could enhance the aroma of dealcoholized Chardonnay wine, aiming to tackle the common sensory drawbacks of dealcoholized wine beverages. Results showed that these extracts significantly enriched the wine’s aromatic profile, marked by increased levels of alcohols, esters, and terpenes, all while keeping the physicochemical properties intact. This research not only offers new strategies for improving non-alcoholic wines but also promises to drive innovation in the beverage industry (Ma and Sam, 2024). In the pharmaceutical field, Baihe Zhimu Decoction (LBRAD), which contains *Lilium* lancifolium bulb and Anemarrhena asphodeloides rhizome, is the first prescription for “Lily Disease” recorded in ancient medical texts. It is also a specific remedy for “Lily Disease” following sweating. The classic LBRAD recipe combines fresh *Lilium* lancifolium bulbs and dried Anemarrhena asphodeloides rhizome slices. It is known for supplementing nutrition, clearing heat, nourishing Yin, and providing moisture (Pan et al., 2024).

In other fields, the sulfuric acid acidification method has been used to make periodic mesoporous organosilica hydrophilic microspheres. PMO aqueous microspheres were made by a co-condensation reaction, using sulfated Lentinus edodes polysaccharide-bridged silane (SLLTPBS) and polyhedral oligomeric silsesquioxane (POSS) as silicon sources. This method solves the problem of using large amounts of acetonitrile in hydrophilic interaction liquid chromatography (HILIC) columns. Compared to ordinary C18 columns, the PMO (SLLTP-POSS) column has much better acid and alkali resistance. At the same time, this stationary phase shows high separation efficiency and selectivity for mixtures of organic acids, sugars, sugar alcohols, amino acids, and sweeteners (Chen et al., 2023). Lily-related cosmetics offer a wide array of functions such as skin care, whitening, antioxidation, anti-aging, wrinkle removal, spot fading, acne treatment, moisturizing, anti-radiation, skin repair, heat clearing, as well as promotion of hair growth and hair blackening. *Lilium* spp. plants are rich in health-beneficial metabolites. Phenolic acids and flavonoids contained in these plants exhibit strong antioxidant, anti-ultraviolet radiation and antibacterial properties. Polysaccharides possess excellent skin moisturizing, antibacterial and skin-repairing capabilities. Other metabolites such as saponins, alkaloids, carotenoids and anthocyanins also offer potential health benefits. These metabolic metabolites provide the material and functional basis for the application of lilies in cosmetics (Tang et al., 2022). Bulb macerates were obtained using 70% and 96% ethanol. The total phenolic content (TPC), total flavonoid content (TFC), condensed tannins (CTC), mineral composition, and antioxidant activity (DPPH assay) were evaluated. Additionally, spectroscopic (FTIR) and antibacterial analyses were performed to further characterize the bulb macerates. Stable hydrogel formulations containing LD-70 and LA-70 were developed, showing excellent pH stability, favorable rheological properties, and sustained antioxidant activity over 60 days. These results support the use of lily-derived macerates in dermocosmetic formulations for skin protection and microbial defense (Lupşor et al., 2025). Yeast-derived biosurfactants, prized for their low toxicity, unique action, and versatility, have broad applications in food, medicine, and cosmeceuticals. We isolated a highly surface-active yeast strain, L3-GPY, from *Lilium* lancifolium Thunb., and identified it as *Aureobasidium* pullulans. From its culture supernatant, we extracted glycerol-liamocin, a novel low-surface-tension metabolite with potent biosurfactant activity (31 mN/m). Our findings indicate glycerol-liamocin holds great promise as a new biosurfactant for various industrial uses (Kim et al., 2015).

**FIGURE 8:**
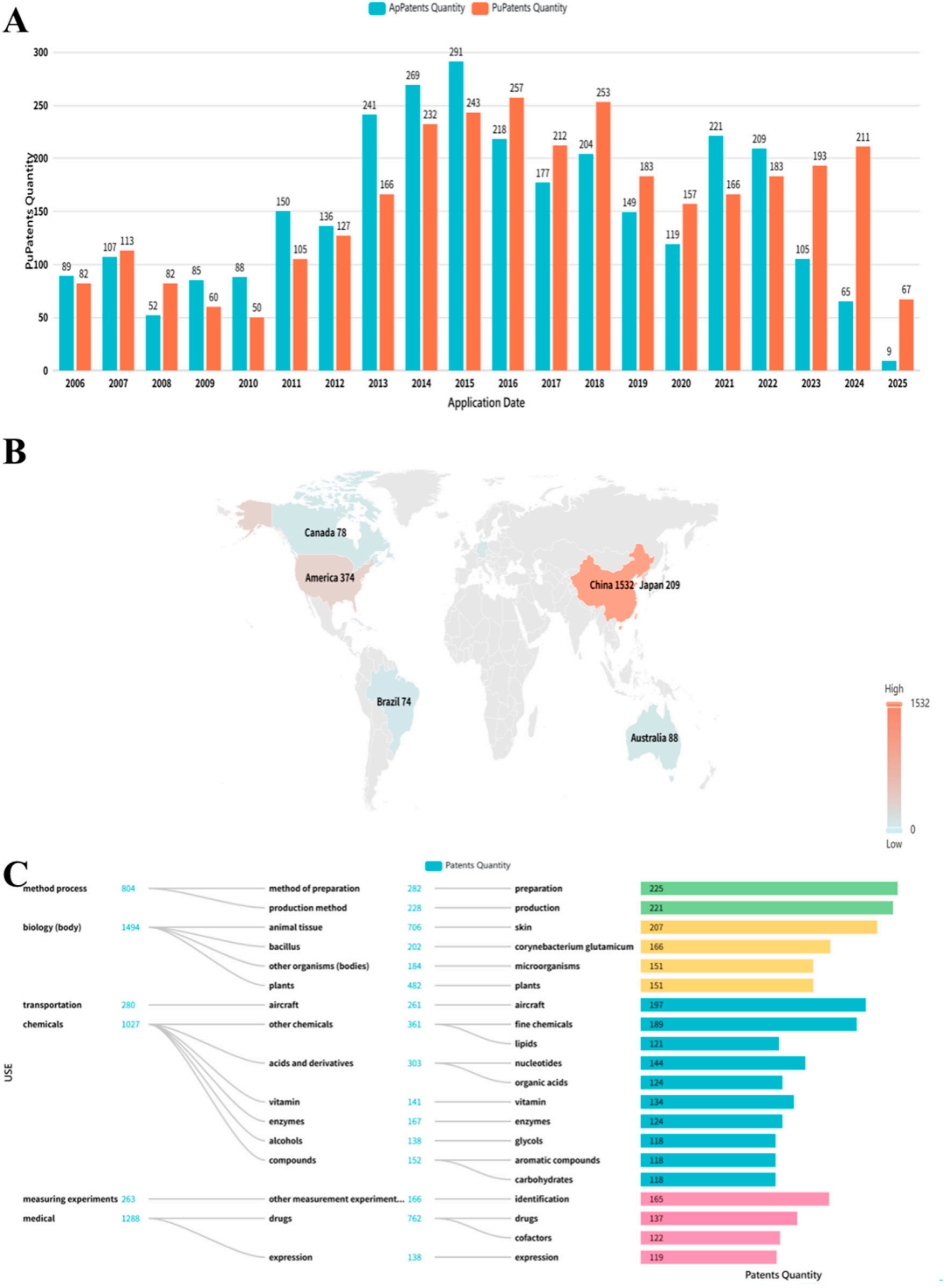
Current situation of patent related to *Lilium* spp. **(A)** Document numbers; **(B)** Patents distribution; **(C)** Patents use.

**FIGURE 9:**
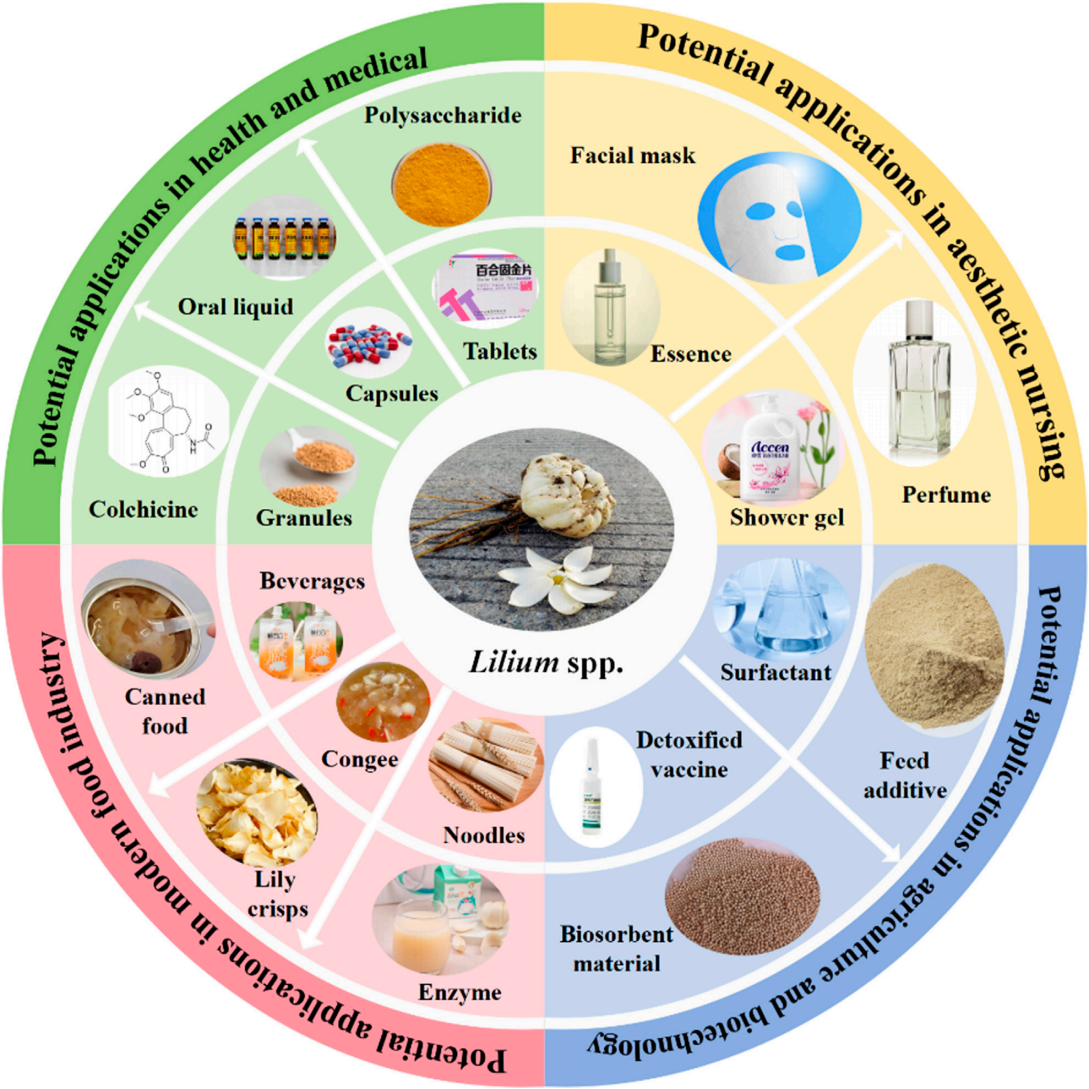
Potential applications of *Lilium spp*. in health and medicine.

Patent activity related to *Lilium* spp. is experiencing significant growth. Current Status of Patent Filings Related to the Genus *Lilium*, with Patent Utilization Illustrated in Figure 8 and Table 4, the past 2 decades have witnessed a substantial increase in patent applications, reflecting growing interest in its potential. To date, **2,517** patents concerning *Lilium* spp. have been granted globally, with **211** issued in **2024** alone. These patents originate primarily from China, the United States, Brazil, Canada, and Australia. The technological scope of these patents is diverse, encompassing preparation and production methods, applications involving animal tissues, and uses with *Bacillus*. Primarily, the reported application potential focuses on areas such as antidiabetic, antidepressant, anti-insomnia, and analgesic effects, alongside immune enhancement, lung-moistening and cough-relieving properties, and health food applications. Table 4 summarizes a list of specific *Lilium* spp.-containing product patents and their claimed biological properties. As research delves deeper into the unique nutritional and medicinal properties of *Lilium* spp., the application of related functional products is expanding. This trend exhibits diversification, broadening the market to better cater to diverse population needs. Consequently, *Lilium* spp. is playing a significant role in pharmaceuticals and nutritional health products. The applications of *Lilium* spp. extend to every aspect of our lives, potential applications in health and medicine are shown in Figure 9.

**TABLE 4:** Patents list of products containing the *Lilium* spp. and their claimed beneficial effects for improving human health.

Application	Main composition	Pharmacological properties	Publish number
Pharmaceutical	Poria, lily bulb, *Gordon euryale* seed, lotus seed, wolfberry, black date, and vine tea	Antidiabetic	CN120392904A
Pharmaceutical	Lily bulb powder	Improve sleep	CN120392948A
Pharmaceutical	Poria, *Radix Pseudostellariae*, *Semen Euryales*, *Radix Puerariae Lobatae*, Celery, *Pericarpium Citri Reticulatae*, *Capsella bursa-pastoris*, *Fructus Crataegi*, Luffa, *Bulbus Lilii*	Enhance pancreatic function	CN120360258A
Pharmaceutical	*Zaocys dhumnades*, *Spatholobus suberectus* Dunn, *Astragalus propinquus* Schischkin, *Angelica sinensis* (Oliv.) Diels, *Scutellaria barbata* D. Don, *Lilium brownii* F.E. Brown var. *viridulum* Baker, *Trichosanthes* peel, and *Platycladus orientalis* (L.) Franco	Analgesia	CN120324536A
Pharmaceutical	Lilial A	Anti-inflammatory	CN117105946B
Pharmaceutical	Lily polysaccharide	Anti-enteritis	CN116650517B
Pharmaceutical	*Radix Ophiopogonis*, *Ginseng Radix et Rhizoma*, *Fructus Mume*, *Bulbus Lilii*, and *Rhizoma Phragmitis*	Antidiabetic	CN120204326A
Pharmaceutical	Lily, lotus seed	Antidiabetic	CN120053556A
Pharmaceutical	Concentrated pear juice, lily bulb, *Polygonatum odoratum*, *Siraitia grosvenorii* fruit, almond, *Semen Sterculiae Lychnophorae*, *Flos Lonicerae Japonicae*, *Platycodon grandiflorus* root, *Chrysanthemum morifolium* flower, tangerine peel, Chinese date, *Polygonatum sibiricum* rhizome, loquat leaf, *Anemarrhena asphodeloides* rhizome, *Gnaphalium affine*, crystal sugar	Moisten the lung and relieve cough	CN120053558A
Pharmaceutical	Lily, *Coptis chinensis*, cinnamon	Anti-heart failure	CN119770577A
Pharmaceutical	*Lilium* longiflorum bulbifera polysaccharide	Immunological activity	CN119638862A
Pharmaceutical	*Rhizoma Coptidis*, *Prepared Fructus Evodiae*, *Cortex Cinnamomi*, *Bulbus Lilii*, *Sclerotium Poriae Paradicis*, *Rhizoma Acori Tatarinowii*, *Prepared Radix Polygalae*, and *Mentha*	Anti-insomnia	CN119157965A
Health product	American ginseng slices, lily bulb, wolfberry, *Polygonatum sibiricum*, peppermint, and jasmine green tea	Invigorate	CN120323540A
Health product	Pueraria powder, natto powder, turmeric powder, inulin, lily bulb powder, *Semen Ziziphi Spinosae* powder, and *Poria cocos* powder	Antidepressant	CN120304514A
Health product	Lotus Seeds, Coix Seeds, Erythritol, Lilies	Sedative	CN120113771A
Health product	*Fructus Canarii*, *Fructus Canarii*, *Semen Sterculiae Lychnophorae*, *Radix Glycyrrhizae*, *Bulbus Lilii*, and *Mentha Haplocalyx*	Soothing the throat and moistening the lungs	CN119867229A
Ecological conservation	Lily Bulb	Water purification	CN120328738A
Plant research	Carotenoid	Regulation of lily colour	CN119685353A

## 8 Future outlooks

This review synthesizes recent advances in *Lilium* spp. research, integrating botanical characterization, ethnobotanical records, nutritional value, phytochemical profiles, pharmacological activities, patent landscapes, and commercial applications. Bulbs of the genus, rich in edible starch, have long served as both food and medicine, while selected species additionally provide aromatic oils for perfumery. The growing recognition of their nutritional assets and broad health benefits has intensified interest from the pharmaceutical and nutraceutical sectors. To date, 123 distinct metabolites have been identified, comprising 68 saponins, 23 phenolics, 13 phenylpropanoids, 17 polysaccharides, and 2 additional metabolites. Among these, saponins, phenolics, and phenylpropanoids underpin a spectrum of bioactivities, including hypoglycaemic, anti-obesity, anti-inflammatory, antioxidant, antitumour, antidepressant, sleep-promoting, and immunomodulatory effects. Accumulating evidence positions *Lilium* spp. as a promising therapeutic resource for depression, insomnia, diabetes, and disorders driven by inflammation and oxidative stress. Collectively, the genus emerges as a dual-purpose functional food and medicinal plant with considerable health-promoting potential and expanding commercial relevance.

Despite the growing body of evidence, several critical knowledge gaps continue to impede the translation of *Lilium* spp. bioactivities into health-promoting applications: (1) Although numerous *in vitro* and *in vivo* studies have demonstrated the efficacy of *Lilium* spp. extracts, the specific metabolites responsible for these effects remain largely unidentified. Systematic isolation, structural elucidation, and mechanism-of-action studies of active monomers are therefore urgently required. Such investigations will clarify structure–activity relationships, enable precise dosing, and accelerate the discovery of novel bioactive scaffolds; (2) Over the past 2 decades, *Lilium* spp. preparations have shown promise in preventing depression, insomnia, and diabetes, as well as in promoting general health. Historical and documentary records also indicate their use as functional foods for managing depression, diabetes, tumors, pulmonary disorders, and other oxidative stress–related chronic diseases. Future research should thus prioritize the development of standardized *Lilium*-based products for dietary therapy, medicine, and cosmetology; (3) Despite centuries of culinary and medicinal use, the nutritional composition and patent landscape of *Lilium* spp. remain insufficiently explored. The bulbs are rich in polysaccharides, saponins, vitamins (B_1_, B_2_, B_3_, and C), amino acids, starch, calcium, iron, and other micronutrients. In traditional Chinese medicine, the bulbs are believed to resolve phlegm, relieve cough, nourish Yin, and moisten the lungs, whereas modern studies emphasize their antioxidant, immunomodulatory, and antihyperglycemic properties. These attributes highlight the potential of *Lilium* spp. as a dual-purpose functional food and pharmaceutical resource, warranting expanded nutritional and intellectual property investigations; (4) Finally, before pharmaceutical formulation can proceed, comprehensive studies of the pharmacokinetic properties of bioactive metabolites—including absorption, distribution, metabolism, and excretion (ADME) processes—are required to elucidate their *in vivo* behavior and inform toxicity assessment. Concurrently, detailed evaluations of both acute and chronic toxicity, as well as analyses of metabolite-related biological effects, are essential to ensure drug safety and efficacy. Moreover, pharmacokinetic insights can facilitate the rational design of dosing regimens and guide the structural optimization of active substances, thereby enhancing therapeutic efficacy and minimizing toxicity.

In conclusion, *Lilium* spp. have drawn broad attention for their plentiful benefits, showcasing significant potential in functional foods, modern medicine, and cosmetics. They serve as a bridge for the internationalization of traditional Chinese medicine, providing developed nations with innovative pathways to enhance human health and contribute to a shared future via modern medical advancements. Despite facing opportunities and challenges in development and applications, *Lilium* spp. are expected to hold vast market prospects in medicine, functional foods, and cosmetics.

## Glossary

AELBB: aqueous extract of *L. brownii* Bulbs
AELDB: Acetone extract of *L. davidii* Bulbs
AI: atherogenic index
AIP: atherogenic index of plasma
AKP: alkaline phosphatase
ALLB: aqueous extract from the *L. lancifolium* Bulbus
ALLF: aqueous extract from the *L. lancifolium* flowers
ALLL: aqueous extract from the *L. lancifolium* leaves
ALLR: aqueous extract from the *L. lancifolium* roots
ALT: alanine aminotransferase
ALP: alkaline phosphatase
AMPK α: AMP-activated protein kinase α
Ang II: angiotensin II
AST: aspartate aminotransferase
BAMBI: BMP and activin membrane-bound inhibitor
Bax: Bcl-associated X protein
BUN: blood urea nitrogen
CAT: catalase
C/EBPα: CCAAT-enhancer binding protein α
COX2: cyclooxygenase-2
Col Ⅲ: collagen type Ⅲ
Col Ⅳ: collagen type Ⅳ
cAMP: cyclic adenosine monophosphate
CTGF: connective tissue growth factor
CVF: collagen volume fraction
CVDs: cardiovascular diseases
CysC: cystatin C
DENA: diethylnitrosamine
DMDD: 2-dodecyl-6-methoxycyclohexa-2,5-diene-1,4-dione
DPPH: 2,2-diphenyl-1-picrylhydrazyl radical scavenging activity
ECE: endothelin-converting enzyme
EELBB: Ethanol extract of *Lilium brownii* Bulbs
ELLB: Ethanol extract of *L. lancifolium* Bulbs
ELLF: Ethanol extract of *L. lancifolium* flowers
ET-1: endothelin-1
FAS: fatty acid synthase
FBG: fasting blood glucose
FINS: fasting insulin
FFA: free fatty acids
FRAP: ferric reducing antioxidant power
G-6-Pase: glucose-6-phosphatase
GDNF: glial cell-derived neurotrophic factor
GHb: glucosylated hemoglobin
GSH: reduced glutathione
GSH-Px: glutathione peroxidase
HA: hyaluronic acid
HDL-C: high-density lipoprotein cholesterol
Hyp: hydroxyproline
IL-6: interleukin-6
iNOS: inducible NO synthase
Inos: inducible nitric oxide synthase
ISI: insulin sensitivity index
LDL-C: low-density lipoprotein cholesterol
LLLBPS: Longya *Lilium* lipopolysaccharide
LLS: Longya *Lilium* saponins
LN: laminin
LPO: lipid peroxidation
MDA: malondialdehyde
MAPKs: mitogen-activated protein kinases
MELLB: methanol extract of *L. ledebourii* Bulbs
MELDB1: methanol extract of *L. davidii* Bulbs
MELDB2: methanol extract of *L. davidii* Bulbs
MLLB: methanol extract from the *L. lancifolium* Bulbs
MLLR: methanol extract from the *L. lancifolium* roots
MMP-2: matrix metalloproteinase-2
MPO: myeloperoxidase
Myd88: myeloid differentiation factor 88
NEFA: non-esterified fatty acid
NF-κB: nuclear factor-κB
NGF: nerve growth factor
NM: not mentioned
PCNA: proliferating cell nuclear antigen
PELDB: polyethylene glycol extract of *L. davidii* Bulbs
p-AMPK α: phospho-AMPK α
PPAR-α/γ: peroxisome proliferator-activated receptors α/γ
Scr: serum creatinine
SCD1: stearoyl-CoA desaturase 1
SDH: sorbitol dehydrogenase
SGOT: serum glutamate oxaloacetate transaminase
SGPT: serum pyruvate transaminase
SOD: superoxide dismutase
SREBP-1c: sterol regulatory element binding protein-1c
STZ: streptozotocin
TAA: total ascorbic acid
TC: total cholesterol
TEAC: trolox equivalent antioxidant capacity
TGF-β: transforming growth factor-β
TGF-β1: transforming growth factor beta 1
TIMP-1: tissue inhibitor of metalloproteinases-1
TL: total lipids
TLR4: Toll-like receptor four
TLRs: Toll-like receptor family
TNF-α: tumor necrosis factor-α
TNOS: total NO synthase
TQ: triglyceride
TG: triglycerides
VLDL-C: very low-density lipoprotein cholesterol
